# ROS‐Scavenging Multifunctional Microneedle Patch Facilitating Wound Healing

**DOI:** 10.1002/adhm.202501886

**Published:** 2025-08-20

**Authors:** Mahshid Kharaziha, Sahar Salehi, Mahshid Shokri, Seyed Mohsen Ahmadi Tafti, Thomas Scheibel

**Affiliations:** ^1^ Department of Materials Engineering Isfahan University of Technology Isfahan 84156‐83111 Iran; ^2^ Department of Biomaterials Faculty of Engineering Science University of Bayreuth 95447 Bayreuth Germany; ^3^ Colorectal Research Center Imam Khomeini hospital complex Tehran University of Medical Sciences Tehran Iran; ^4^ Division of Colorectal Surgery Department of Surgery Tehran University of Medical Sciences Tehran Iran; ^5^ Bayreuther Zentrum für Kolloide und Grenzflächen (BZKG) University of Bayreuth 95447 Bayreuth Germany; ^6^ Bayreuther Zentrum für Molekulare Biowissenschaften (BZMB) University of Bayreuth 95447 Bayreuth Germany; ^7^ Bayreuther Materialzentrum (BayMAT) University of Bayreuth 95447 Bayreuth Germany; ^8^ Bayerisches Polymerinstitut (BPI) University of Bayreuth 95447 Bayreuth Germany

**Keywords:** arginine functionalized particles, flightless I siRNA, immunomodulatory properties, microneedle patch, ROS scavenging, wound healing

## Abstract

Chronic wounds often experience delayed healing due to a disrupted microenvironment marked by persistent inflammation and excessive fibrosiswhich leads to tissue dysfunction. Excessive mitochondrial reactive oxygen species (ROS) at the injury siteworsen this by hindering healing and promoting scarring. This study presents a multifunctional microneedle array (MNA) that combines ROS scavenging with accelerated tissue formation. The array contains Flightless I (Flii) siRNA‐loaded arginine‐functionalized poly (β‐amino ester)‐alginate (APA) particles within a hyaluronic acid methacrylate (HaMA) matrix. These siRNA‐APA particles enhance ROS scavenging through nitric oxide (NO) delivery and silence Flii expression in HaCaT cells. The synergistic release of NO, arginine, and siRNA reduces pro‐inflammatory gene expression (TNF‐α and IL6), transforming M1 macrophages into the M2 phenotype and aiding the transition from inflammation to proliferation. Studies in a rat chronic wound model demonstrate that applying the siRNA‐APA‐laden MNA transdermally mitigates scar formation and promotes wound healing by reducing inflammatory responses. This siRNA‐APA‐laden HaMA MNA, with ROS scavenging and immunomodulatory activity, offers promising insights into effective chronic wound managementand wound dressing applications.

## Graphical abstract

A reactive oxygen species (ROS) scavenging and immunomodulatory microneedle patch based on hyaluronic acid methacrylate (HaMA) and Flightless I (Flii)‐siRNA‐laden arginine functionalized poly (β‐amino ester)/alginate particles is developed for chronic wound healing applications.

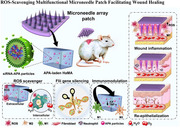

## 1 Introduction

Skin is essential for protecting internal organs from mechanical, chemical, and pathological damages, and it also helps prevent dehydration and infections. Skin disorders and injuries have garnered significant focus owing to their noticeable symptoms, widespread occurrence, and associated death rates.^[^ ^1^ ^]^ The process of wound repair following injury is intricate, involving, numerous cellular and biochemical events aimed at restoring tissue integrity.^[^ ^2^ ^]^ Typically, classical wound healing is clarified through four phases, including hemostasis, inflammation and contractile responses, proliferation, and tissue remodeling. In this context, regulating the immune microenvironment is crucial for this process. When wound healing is impaired due to immunoregulatory dysfunctions, it can lead to chronic wounds characterized by persistent inflammation.^[^ ^3^ ^]^ Such delays can be caused by bacterial infections that disrupt macrophage polarization and trigger excessive secretion of pro‐inflammatory cytokines like tumor necrosis factor‐α (TNF‐α), interleukin‐1 (IL1‐α), and IL6, as well as significant enlargement of mitochondrial reactive oxygen species (ROS).^[^ ^4^ ^]^ While low levels of ROS can enhance angiogenesis and wound healing, excessive ROS can increase oxidative stress, cause mitochondrial dysfunction, prolong inflammation, and delay healing. resulting inpoor skin function and scar formation.^[^ ^5^ ^]^ Accordingly, there is a critical need for precise regulation of ROS levels in the wound microenvironment during various phases of repair, especially for managing chronic woundsin clinical settings.

Various approaches have been investigated so far to overcome chronic wounds by reprogramming mitochondrial activity, facilitating efficient anti‐inflammatory and antioxidative processes in the damaged site. A few examples of these strategies include the administration of anti‐inflammatory drugs,^[^ ^6^ ^]^ applying wound dressings with ROS‐removing efficacy,^[^ ^7^ ^]^ the introduction of naturally derived or synthetic nitric oxide (NO) donors within the wound dressings,^[^ ^8^ ^]^ nanotechnology‐mediated antioxidative therapy,^[^ ^9^ ^]^ small interfering RNA (siRNA),^[^ ^10^ ^]^ and cell therapy.^[^ ^11^ ^]^ In the case of nanotechnology‐mediated therapy, for instance, Zeolitic imidazolate framework‐8 nanoparticle‐bonded cerium dioxide was used which showed a significant decrease in ROS production and reduced cell apoptosis.^[^ ^9^ ^]^ Another interesting research utilized a camouflaged meta‐defensome, which scavenged mitochondrial ROS and inhibited NO synthase. This metabolic reprogramming of mitochondrial activity promoted the repolarization of M1 macrophages into M2.^[^ ^7^ ^]^ In chronic wound healing, NO plays a crucial role in the regulation of immune function and in endorsing wound healing.^[^ ^12^ ^]^ Several studies have concentrated on the controlled release of NO using low molecular weight NO donors, including diazeniumdiolates (NONOates) and S‐nitrosothiols (RSNOs), without the need for enzymes.^[^ ^13^ ^]^ However, by‐products of NONOates are toxic, and the restricted half‐life of RSNO negatively affects their potential application.^[^ ^14^ ^]^ L‐arginine (Arg), recognized as a natural NO donor, is one of the most vital nutritional components and is critical in wound healing. The guanidine group in the molecular structure of the Arg monomer is an alkaline group. The carbon‐nitrogen double bond (C═N) in Arg possesses reductive properties, giving it antioxidant properties. Along with anti‐inflammatory and active participation in metabolic processes, Arg can produce creatine, polyamines, agmatine, and NO, all of which contribute to tissue regeneration.^[^ ^15^ ^]^ Arg could generate NO using the catalysis of inducible NO synthase or through the oxidation of ROS.^[^ ^16^ ^]^ Therefore, Arg could control ROS levels during wound healing.^[^ ^17^ ^]^ Recently, several studies have concentrated on delivering Arg to the wound site in a sustained and targeted manner, which is a strong approach for promoting wound repair.^[^ ^18^ ^]^ We have recently reported the creation of Arg functionalized poly (β‐amino ester) (AP) that, at the optimized conjugation ratio of Arg to Poly (β‐amino ester)(3:1) could form polyplexes with alginate, named APA particles.^[^ ^19^ ^]^ We observed that the controlled release of Arg slightly boosted anti‐inflammatory gene expression. However, the potential of this novel material as an antioxidant and ROS‐scavenging material for biofabrication applications remains to be investigated in vitro and in vivo.

As mentioned above, another promising strategy for wound healing is applying small interfering RNA (siRNA) to silence gene expression.^[^ ^20^ ^]^ This approach has also shown considerable potential in treating fibrosis. Flightless I (Flii) is a part of the gelsolin family of cytoskeletal proteins. It regulates actin dynamics by removing present filaments and/or covering their ends, thereby facilitating the reformation of new cytoskeletal structures.^[^ ^21^ ^]^ It has been reported that Flii is secreted following injury or stimulation by lipopolysaccharide (LPS). It can attenuate Toll‐like Receptor (TLR) inflammatory pathways, thereby decreasing TLR signaling and the production of pro‐inflammatory cytokines.^[^ ^22^ ^]^ In addition, reducing Flii could be promoted in vivo by lowering TLR4 expression and improving angiogenesis.^[^ ^23^ ^]^ Despite Flii's role of Flii in wound healing, there is limited research on using controlled release of Flii siRNA to minimize wound scarring. We have recently showed a controlled release of Flii siRNA from APA particles, leading to a decrease in in vitro Flii gene expression.^[^ ^19^ ^]^ However, it is known that siRNA is vulnerable to breakdown by nucleases present in the bloodstream.^[^ ^24^ ^]^ Therefore, preserving the siRNA stability during its release from particles and systemic circulation is crucial for effective delivery in vivoFurthermore, treatments via efficient delivery of siRNA to specific cells and minimizing off‐target effects are essential for the success of siRNA‐based therapies.^[^ ^25^ ^]^ However, both these treatments are still challenging, as introducing any foreign particles into the body can trigger an immune response and can lead to the clearance of particles, affecting their circulation time.^[^ ^26^ ^]^

To reduce the potential systemic side effects of other treatments, the transdermal approach has offered a direct route for therapeutics to reach damaged areas, bypassing the gastrointestinal tract.^[^ ^27^ ^]^ However, the skin's complex barrier system poses a substantial obstacle to the transportation of active ingredients, thereby constraining therapeutic effectiveness. Furthermore, naked siRNA, having low lipophilicity and high molecular weight, encounters difficulties in permeating the stratum corneum, the outermost layer of the epidermis.^[^ ^25^, ^28^ ^]^ As a result, significant efforts have been dedicated to developing topical siRNA formulations and other large biological molecules. Among them, polymeric microneedle arrays (MNAs) have emerged as efficient tools for transdermal drug delivery by generating micro‐perforations in the stratum corneum.^[^ ^29^ ^]^ MNAs which can penetrate the epidermal layer and anchor the needle tip to prevent detachment while ensuring oxygen access. This makes them a painless, non‐invasive, and infection‐free approach.^[^ ^30^ ^]^ Chun et al.^[^ ^31^ ^]^ developed an MNA patch made of hyaluronic acid (HA) containing nanoplexes of tyramine‐modified gelatin (Gtn‐Tyr) and siRNA targeting secreted protein acidic and cysteine‐rich (siSPARC) for topical scar prevention. In vitro tests with dermal fibroblasts demonstrated that the siSPARC/Gtn‐Tyr nanoplexes and the siSPARC/Gtn‐Tyr‐loaded MNA patches significantly reduced SPARC gene expression without any detectable cytotoxicity. Additionally, the siSPARC/Gtn‐Tyr‐loaded MNA patch decreased collagen assembly and did not trigger any immune response.

**Figure 1:**
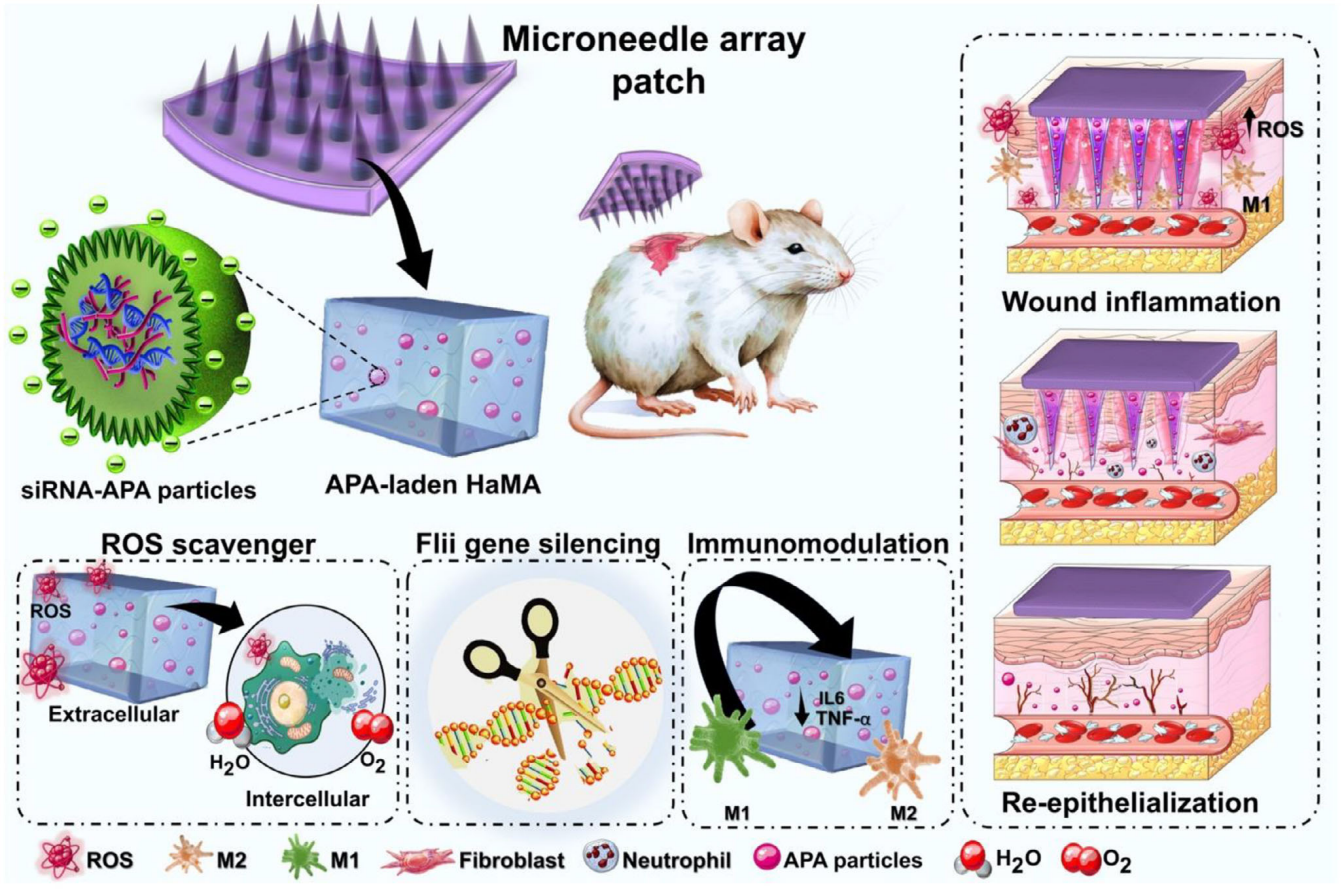
Illustration of the role of microneedle array (MNA) patch based on hyaluronic acid methacrylate (HaMA) incorporated with siRNA encapsulated in Arg functionalized poly (β‐amino ester)‐alginate (APA) particles in chronic wound healing; The release of Flii siRNA‐laden APA could stimulate reactive oxygen species (ROS) scavenging, enhance Flii gene silencing, and induce immunomodulatory properties via promotion of M1 to M2 macrophage phenotype polarization, leading to accelerated wound remodeling.

This research marks a new era in the development of effective immunomodulatory strategies for treating chronic wounds. Furthermore, it offers valuable insights for designing future wound dressings that synergistically incorporate ROS scavenging, anti‐inflammatory, and antioxidation properties, ultimately promoting wound healing. Here, a multifunctional MNA patch based on hyaluronic acid methacrylate (HaMA) incorporated with Flii siRNA‐laden APA particles was developed. **Figure** **1** illustrates the aim of this study in the fabrication and application of multifunctional MNA patches that can release APA particles in the wound, followed by their uptake through the endocytosis process of cells, Arg release, and enhanced chronic wound healing. This process will be via various functions offered by the MNA patches, including Flii siRNA release, anti‐inflammation and antioxidation properties, ROS scavenging, immunomodulation, and the potential for chronic wound regeneration.

## 2 Results and Discussion

### 2.1 Physiochemical Characterization of the MNA Patch

The MNA patches are based on HaMA, incorporated with APA particles. We recently reported the production of APA particles through a simple layer‐by‐layer process using Arg functionalized poly(β‐amino ester) (AP) as the positive layer and negatively charged siRNA. We further optimized these polyplexes’ production with distinct features, such as tunable zeta potential, particle size, and Arg content.^[^ ^19^ ^]^ To further increase the stability of the complex in the environment, the surface of the particles was coated with alginate. The measured zeta potential values show that the presence of siRNA could change the zeta potential of the particles from a positive value to −39 ± 5 mV (Figure S1A, Supporting Information). Moreover, the hydrodynamic diameter of particles decreased from 269 nm to less than 164 nm after interacting with Flii siRNA, owing to the strong interaction between Arg and siRNA. Scanning electron microscopy (SEM) image of these particles (Figure S1B, Supporting Information) reveal the spherical APA particle formation with a relatively uniform size in the range of 200–300 nm.

The MNA patches based on HaMA will benefit from the degradability of HA and the creation of a moist environment for wound healing however, with a controlled degradation rate.^[^ ^32^ ^]^ Findings indicated that the properties of the HaMA hydrogel can be controlled by tuning its crosslinking degree. Therefore, we synthesized HaMA by conjugation between carboxyl groups on HA and methacrylate groups via amide bonds (Figure S2A, Supporting Information) to fabricate the needle‐filling hydrogel (Figure S2A, Supporting Information). By comparing the proton nuclear magnetic resonance (^H^NMR) spectrum of HA (Figure S2B, Supporting Information) and HaMA (Figure S2C, Supporting Information), the presence of the methyl group in the ─CO─C(CH_3_)═CH_2_ of methacrylate was observed at 5.27 and 6.13 ppm, while the signal at 1.96 ppm corresponded to the methyl groups on the HA side chains. This confirmed the successful synthesis of HaMA. The methacrylation degree was calculated to be about 25.3% by integration of methacrylate proton signals at 6.13 and 5.27 ppm, to the peak at 1.96 ppm related to the N‐acetyl glucosamine of HA. This degree of methacrylation was selected based on a comprehensive review of similar studies involving the formation of microneedles using HaMA.^[^ ^33^ ^]^ In other studies, it has been found that higher methacrylation degree did not result in a significant increase in mechanical performance.^[^ ^34^ ^]^ Previous research has demonstrated that this methacrylation degree is an optimized range to provide appropriate mechanical properties, swelling ability, drug release profiles, and biocompatibility for HaMA.^[^ ^33^, ^35^ ^]^ By evaluating these findings, we aimed to use the optimized methacrylation degree to achieve the desired balance between properties, ensuring effective skin penetration and controlled drug delivery.

**Figure 2:**
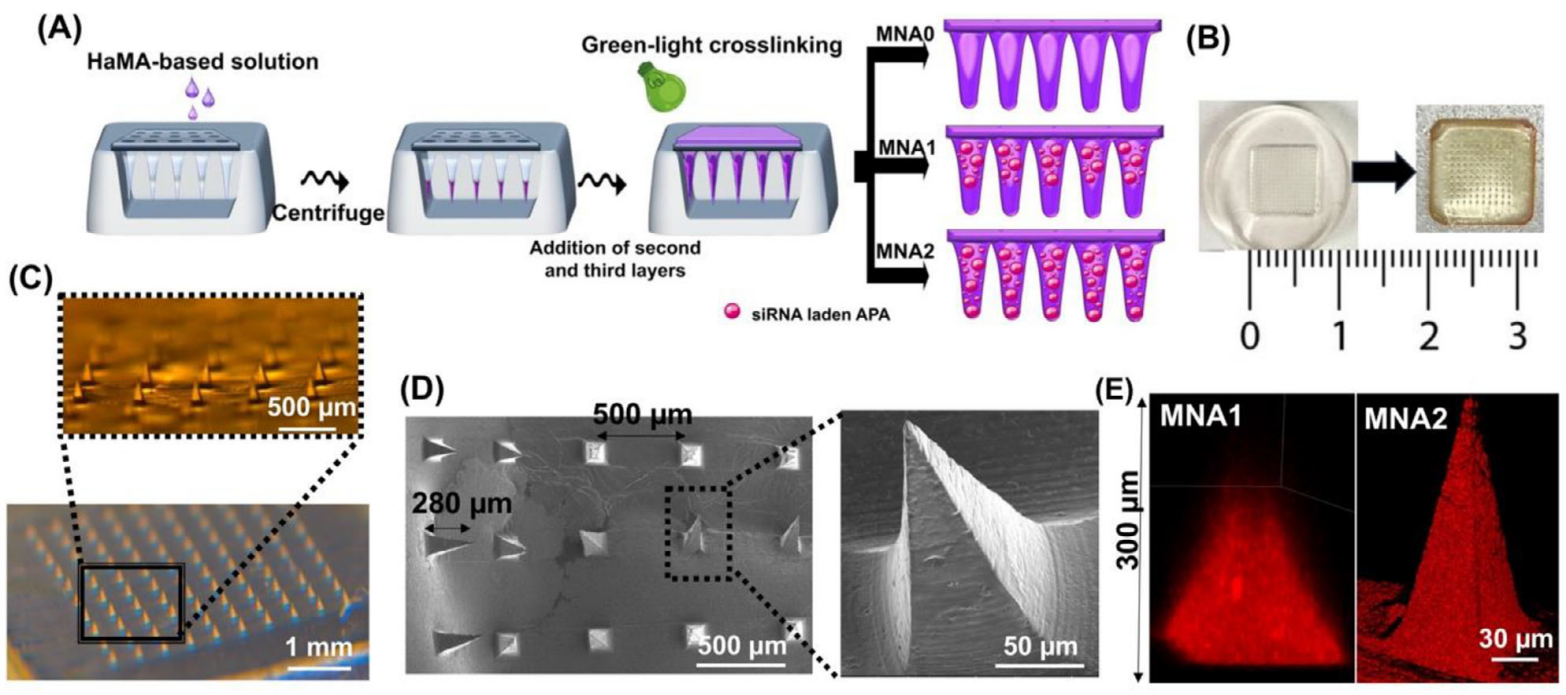
Fabrication and characterization of the multifunctional MNA patches: A) illustration of the fabrication process of the multilayered structure of MNA patches. B) Digital microscopy images of the PDMS mold and MNA patches. C) The representative stereomicroscope images of the MNA patch (scale bars: 500 µm and 1 mm), D) SEM images (scale bars: 500 and 50 µm), and E) 3D confocal image of Rhodamine B‐labelled siRNA‐APA incorporated MNA patches (Red: Rhodamine B, scale bar: 30 µm). F) MNAs with stack images showing the layer spacing of 50, 150, 200, and 250 µm within the height of the MNAs (scale bar: 250 µm).

Next, following the procedure shown in **Figure** **2**A, the biodegradable HaMA‐based MNA patches are fabricated using a layer‐by‐layer process to ensure that the polyplexes are loaded at the MNAs using a polydimethylsiloxane (PDMS) microneedle mold (Figure 2B). Figure 2C,D, the representative stereomicroscopic and SEM images show the microneedle molds arranged in a 10 × 10 array, including 100 needles with a distance between the two tips of 500 µm, with identical conical needles (height: 280 µm and base diameter: 150 µm), which enable the quick penetration of microneedles into the stratum corneum of the skin and the effective delivery of drugs in the wound.^[^ ^36^ ^]^ A reduction in height compared to the respective master structures might be due to the volumetric contraction during drying. According to Figure 2A, to control siRNA release, a layer‐by‐layer coating strategy is performed with different compositions in each layer, leading to the formation of three different MNA patches; MNA0, MNA1, and MNA2, containing no siRNA‐laden APA particles in the HaMA layer, only in the second layer, or in both first two layers, respectively. When examined under a fluorescence microscope (Figure 2E), the Rhodamine‐labeled siRNA (red color)‐laden APA particles were distributed within MNA patches, depending on the designed patches. While siRNA‐laden APA particles in HaMA were deposited as the second layer in MNA1, in MNA2, the particles were loaded in both the first layers. The backing substrate layer in MNA patches was made of pure HaMA. Therefore, we have shown that MNA loaded with siRNA‐laden APA particles was synthesized, which would potentially permit transdermal delivery. The continuous fluorescence signals from the dye at various depths are observed in Figure 2F. Red fluorescence signals were detectable from the surface to the deeper layers. Additionally, it was observed that the fluorescence area gradually diminished with increasing depth during scanning.

The Fourier Transform Infrared (FTIR) spectra of samples in Figure S3 (Supporting Information) confirmed the successful loading of siRNA‐laden APA particles in HaMA‐based MNA patches. FTIR spectrum of HaMA consists of a broad peak at 3200–3500 cm^−1^ related to the O─H stretching, indicating the hydrogel‐bond hydroxyl groups. The peaks identified at 2850–2920, 1610–1630, and 1550–1580 cm^−1^ correspond to the C─H and C═O stretching as well as N─H bending of amide II, respectively. The ether linkage in the polysaccharide backbone is also determined according to the C─O─C stretching at 1000–1150 cm^−1^. The sharp peak at 1720–1740 cm^−1^, associated with the ester carbonyl group, and C═C stretching at 1630–1640 cm^−1^ are the characteristic peaks confirming the methacrylation of the HA backbone.^[^ ^37^ ^]^ In Figure S3 (Supporting Information), the FTIR spectrum of the siRNA‐laden APA particles consists of the main peaks related to Arg, Alginate, and poly(β‐amino ester) matrix. The C─O band identified at 1181 cm^−1^, C═O stretching at 1700–1750 cm^−1^, aliphatic C─H stretching vibration in the region of 2850–3000 cm^−1^, N─H stretching vibration at 1650–1700 cm^−1^, and N─H stretching vibration in 3200–3500 cm^−1^ are related to the poly (β‐amino ester) (PAE) matrix. Some of the peaks overlap with the characteristic peaks of Arg and alginate. For instance, the amide I band arising from the carbonyl (C═O) stretching vibrations in the Arg chain and carboxylate band of alginate could also be identified at 1600–1700 cm^−1^. Moreover, the C─N bond of the Arg side chain and the C─O─C stretching vibration of the glycosidic bond are identified in the region of 1000–1100 cm^−1^. Furthermore, the C─O─H bending vibration of the hydroxyl groups and the C─C stretching vibration of the aliphatic groups are observed in the region of 1300–1500 and 1000–1200 cm^−1^, respectively. After the formation of the MNA1 patch, the peaks related to both components could be identified, confirming the successful formation of the hybrid MNA patch without any structural distraction.

### 2.2 Mechanical Performances of MNA Patches

**Figure 3:**
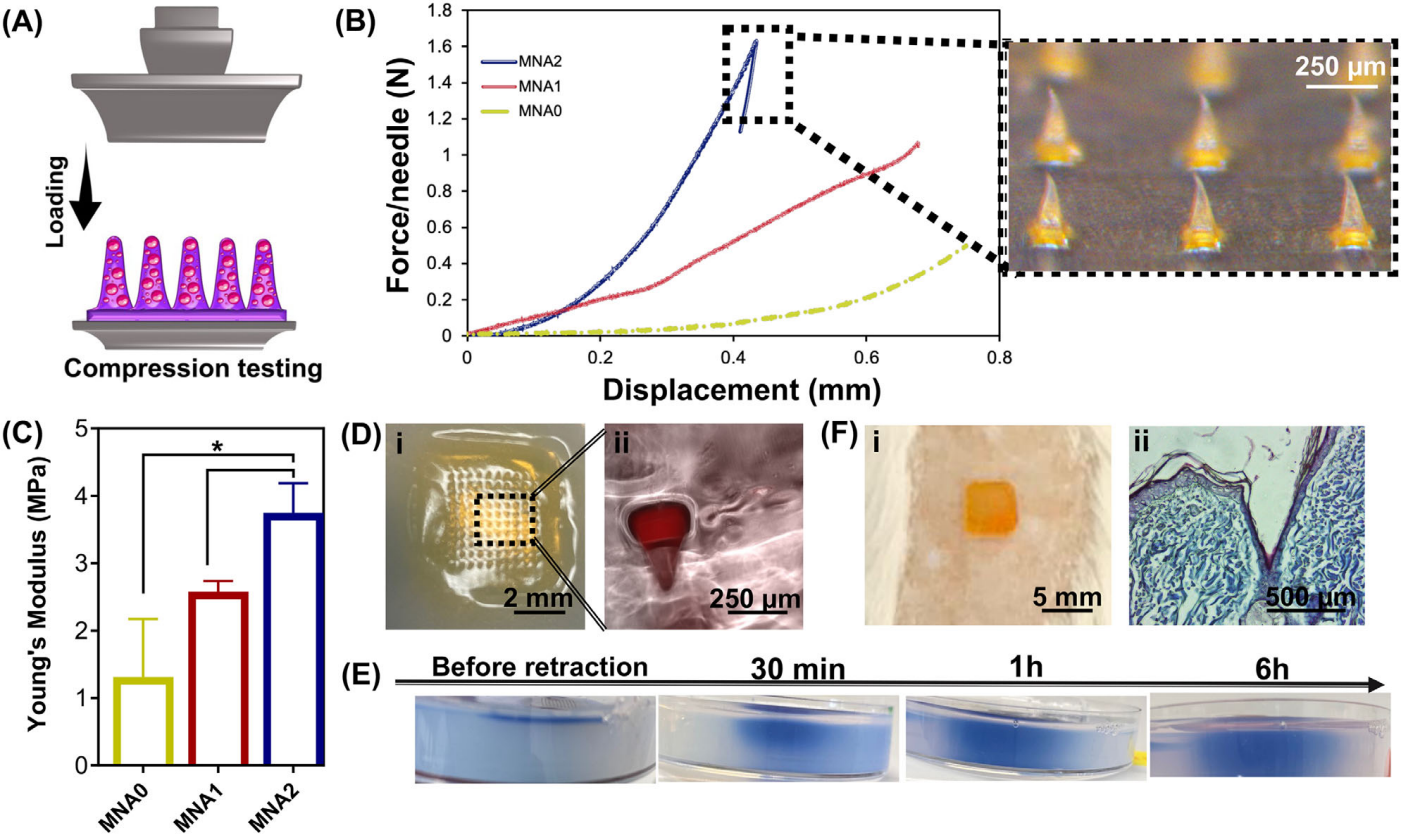
Mechanical performance of MNA patches: A) The schematic showing the setup of the compression test applied on MNA patches, B) the force–displacement graph of MNA patches with varying compositions in layers (MNA0, MNA1, and MNA2) as well as the bright‐field images of the MNA1 patch after the compression test taken by stereomicroscope, C) The average compressive modulus of MNA patches, extracted from compression tests (*P < 0.05), D) In vitro agarose gel puncture characterization of the MNA patch: i) Photographs of the MNA patch administered into the agarose gel and ii) A fluorescent image of the Rhodamine B‐loaded MNA patch penetrating into the agarose gel, E) Photographs of the agarose gel during the penetration of methylene blue (MB) loaded‐MNA1 and removing after different time points, F) *Ex vivo* rat skin puncture characterization of the MNA patch: i) Digital image of MNA1 patch administered into a rat's cadaver skin and ii) Masson's trichrome‐stained cadaver skin showing a microhole created by the MNA1.

To confirm if the fabricated MNA patches can successfully penetrate the skin tissue without damaging the needle shape and compromising their function (specifically, the effective delivery of siRNA‐APA particles to the dermis), the mechanical performances of the MNAs were characterized using a universal testing machine. Accordingly, the compression performances of MNAs were tested using a standard compression setup comprising two stainless‐steel plates applying force to MNAs, as shown in **Figure** **3**A. Subsequently, the acquired force and displacement parameters were plotted to generate a force–displacement graph. The profiles of compression force displacement in Figure 3B show that the force–displacement curves experienced a sudden drop upon reaching the force saturation point. In both samples, the highest load applied was regarded as the failure load. Furthermore, the compressive force of the MNAs was enhanced by increasing the content of APA particles in them (MNA2). The MNA imaged after the compression test is shown in Figure 3B, indicating the absence of discontinuous points. that can be interpreted in a way that MNA is rather bent than being shattered, crushed, or broken off from the substrate under stress. Accordingly, the average forces required to bend MNA1 and MNA2 were 1.2 and 1.6 N on the needle, respectively, which are higher than those for MNA0 (0.6 N on the needle). This indicates that these patches will have adequate mechanical strength for penetrating the skin.^[^ ^38^ ^]^ Moreover, compared to other MNA patches based on GelMA containing silicate nanoplatelets (≈54 ± 4 mN on the needle^[^ ^39^ ^]^), HA incorporated with various nanoparticles (≈0.7–1 N on the needle^[^ ^40^ ^]^), chitosan (>1.5 N on the needle^[^ ^41^ ^]^) and polycaprolactone (PCL)/polylactic‐*co*‐glycolic acid (PLGA) polymers (≈0.3–0.8 N on the needle^[^ ^39^, ^42^ ^]^), our patches revealed improved or comparable mechanical performances. In addition, since the failure force exceeded the minimum force (≈0.2 to 3 N on the needle) found in the literature^[^ ^43^ ^]^ necessary to breach the stratum corneum layer, we can conclude that the MNAs are sufficiently durable to penetrate soft tissues without fracturing. However, we also found that the presence of APA loading in both first layers of needles significantly affected the mechanical performances of MNA patches, as the same force deformed the MNA1 and MNA2 patches, resulting in different displacements. We further quantified the compressive modulus of MNAs, Figure 3C, showing an increasing trend as the APA concentration increased up to two layers in MNA2. Materials with higher compressive modulus showed a failure load at lower displacement during compression tests. Our results showed that the compressive modulus of the MNAs was remarkably increased to 3.6 ± 0.3 MPa in the MNA2 sample. This value was significantly improved compared to the compressive modulus of most of the MANs patches reported before,^[^ ^44^ ^]^ which might be due to the efficient role of chemical interactions formed between the HaMA matrix and APA particles. Consequently, based on the data presented, the prepared MNAs may be suitable for puncturing and inserting into skin tissue.

To verify the penetration of the MNA patch, preliminary indentation experiments were conducted using agarose gel to evaluate the insertion capabilities of the MNA1 patch containing Rhodamine B (red)‐labeled siRNA‐laden APA particles (Figure 3D‐i). Agarose gel is an ideal testing medium due to its tunable mechanical properties and transparency, enabling real‐time microneedle penetration observation.^[^ ^45^ ^]^ The experiments demonstrated that Rhodamine B‐labeled particles were uniformly distributed in the MNA1 patch, and microneedles ≈300 µm in height could penetrate the agarose gel to a depth matching the height of the microneedle cones (Figure 3D‐ii). Importantly, no breakage or bending of the microneedle was observed during these insertion tests, indicating their structural integrity and potential suitability for practical applications. Next, the methylene blue (MB, a cationic dye)‐filled MNAs were inspected after penetration into the agarose gel. The images from the MNA1 patch, after retraction from the agarose gel, at different time points of 30 min, 1 h, and 6 h, are shown in Figure 3E. It can be seen that the blue color remained intact during microneedle insertion and after retraction. Upon needle removal, during 6 h, the spatial distribution of blue color within the gel shows that the rate of dye diffusion was slow (Figure 3E). We also performed *ex vivo* insertion testing after penetration of the MNA patch in a rat cadaver skin model and removed it. Shortly before the MNA was applied to the skin, the surface of the tissue was wetted with a saline solution. Figure 3F‐i shows that the patch easily penetrated the rat skin with the application of finger pressure and remained there owing to the swelling of the MNA patch upon absorbing interstitial fluid, which created a mechanical interlocking effect. The histology images of Masson trichrome staining of the skin (Figure 3F‐ii) showed the successful penetration of MNA through all layers of the epithelium and into part of the dermis. The needle‐created holes were distinctly visible, extending from the epidermis into the dermis, given the image, that the MNA patch established a transdermal drug delivery channel without compromising the integrity of the dermis. Therefore, it could be confirmed that the MNA patches could have the potential for use in drug delivery applications. The observed discrepancy between the insertion depth and the actual length of the MNA was due to the deformation of the viscoelastic skin surface and manual errors made while cutting the skin during sectioning.

### 2.3 Physiological Performances of MNA Patches

**Figure 4:**
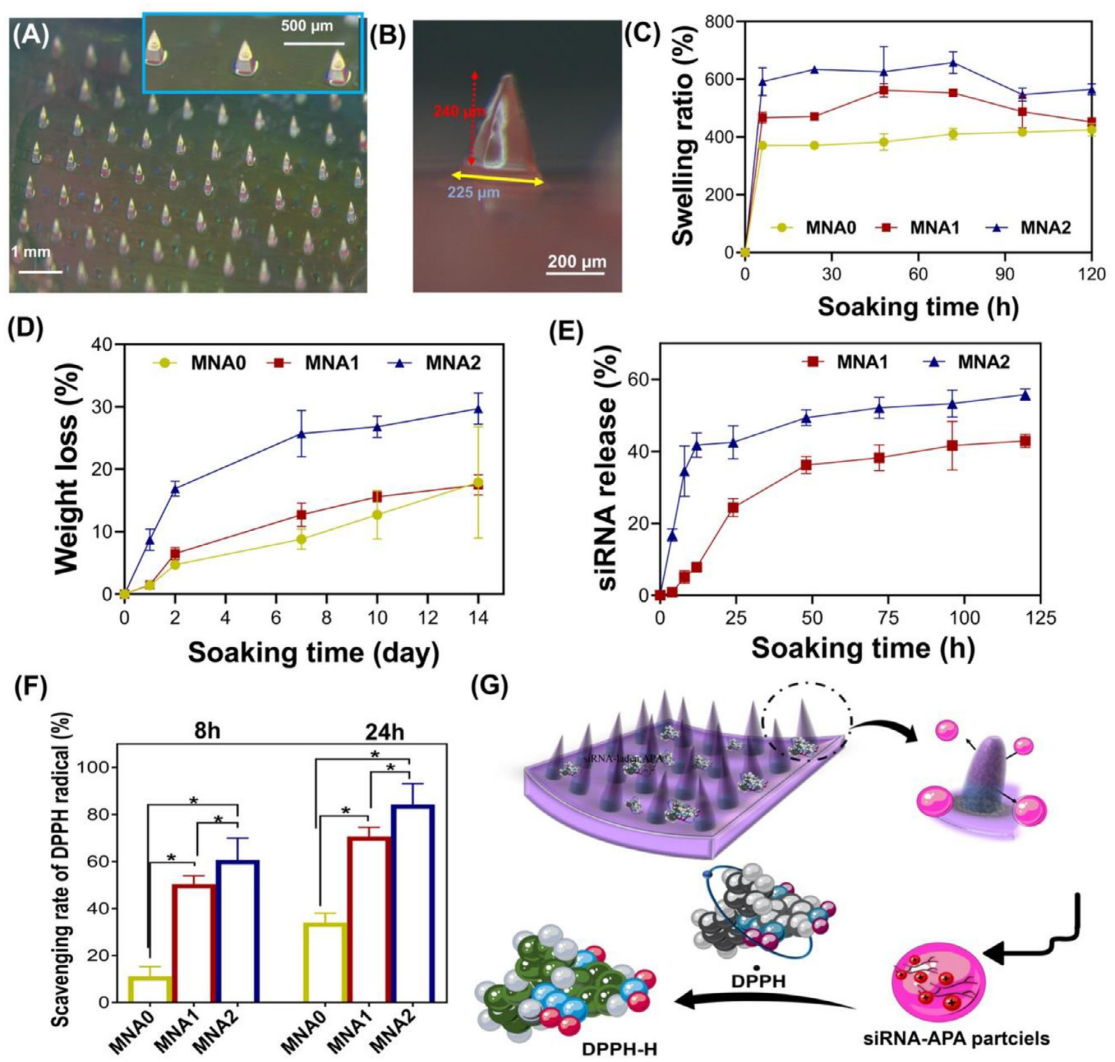
Physiological stability of MNA patches and sustained siRNA release: Stereomicroscope images of A) a MNA patch and B) a needle of this patch after soaking in PBS for 24 h. C) The swelling ratio of MNA patches during 120 h of soaking in PBS at 37 °C. D) The weight loss of MNA patches during 14 days of immersing in PBS containing 5 U mL^−1^ of HAase at 37 °C. E) siRNA release from siRNA‐APA‐laden MNA patches incubated in PBS at 37 °C for 120 h. F) ROS scavenging property of MNA patches against 1‐diphenyl‐2‐picrylhydrazyl (DPPH) radicals: scavenging rate of DPPH radicals (%) after 8 and 24 h incubation (*P < 0.05). G) The schematic illustration of the antioxidant property of MNA patches.

To investigate the swelling rate and degradability of the MNA patches, the samples were immersed in phosphate‐buffered saline (PBS) at 37 °C and pH = 7.4. The bright‐field images of the MNA1 sample after 24 h soaking in PBS and its geometry are presented in **Figure** **4**A,B. The height of the microneedles reduced, and they expanded during soaking in PBS, while they preserved their structure owing to the strong chemical bonding in the HaMA composition. This stable shape supports the mechanical interlocking of MNA patches after in vivo skin penetration. The swelling kinetics study showed a rapid increase in the sample's weight during the first 10 h, after which the swelling rate reached a stable state over time (Figure 4C). As the amount of APA increased, the equilibrium swelling rate of the MNAs increased. For instance, after 24 h, the equilibrium swelling rate increased from 370 ± 28% (at MNA0) to 634 ± 84% (at MNA2). Within the hybrid systems, the electrostatically induced hydration due to the hydrophilic nature of APA particles promoted the hydrogel network's expansion, elucidating the comparatively high expansion rate of the hydrogels. The hygroscopicity of MNA and its porous structure could create a conducive environment for wound healing by absorbing excessive tissue‐penetrating fluids produced on the wound surface. The porous structure of HaMA hydrogel supports the uptake and delivery of molecules at a controlled rate by varying their pore sizes. The weight loss curve of MNA patches (Figure 4D) presents a high degradation rate within the first 2 days of incubation, before it becomes slower in later stages. The decreased crosslink density in MNA2 and less binding between molecular chains of HaMA can be interpreted as increased weight loss and higher water absorption compared to MNA0 and MNA1. These behavioral changes were attributed to the presence of APA particles with high amounts of amide bonds in their molecular structure, which made them hydrophilic and accessible to hydrolysis. The difference in the degradation rate between MNA1 and MNA2 patches was also visible after comparing the SEM images of both patches after 14 days of incubation in PBS containing 5 U mL^−1^ of HAase (Figure S4, Supporting Information). The MNA1 patch and the needle shape remained intact compared to MNA2, which appeared fully degraded. For later application in skin regeneration in vivo, the type of MNA and their stability can define which type of patches should be applied, as it also controls the release rate of APA particles and, consequently, the release rate of the siRNA in the wound site.

To mimic the biological environment, siRNA release was investigated in PBS for 5 days. As depicted in Figure 4E, the measured siRNA release profile displayed a sustained release, depending on the composition of MNA. Following the initial burst release, the MNA1 and MNA2 patches exhibited a slow release and reached 24.4 ± 2.3% and 42.5 ± 4.5% for MNA1 and MNA2, respectively, after 60 h incubation. It could be reported that, following APA release due to the swelling ability of MNA patches, APA particles were released and hydrolyzed, leading to degradation of bonds in the APA particles and siRNA release.^[^ ^46^ ^]^ According to this hypothesis, the potential antioxidant effects of the MNA patches were assessed by 1‐diphenyl‐2‐picrylhydrazyl (DPPH) radical‐scavenging assay.^[^ ^47^ ^]^ The color changes in the DPPH solution during the soaking of MNA patches are shown in Figure S5 (Supporting Information), indicating a dose‐dependent ROS‐scavenging activity owing to the available APA particles. While the MNA0 group did not affect free‐radical species, a deep purple color of pure DPPH (0.5 mm) disappeared with increasing incubation time from 8 to 24 h and APA content, revealing the enhanced antioxidant property as a result of APA release. The changes in the color of DPPH were quantified via UV–vis spectroscopy. Our results showed that the scavenging ability of DPPH depended on the increased concentrations of APA particles, reaching 58 ± 9% after 8 h for MNA2 samples (Figure 4F). Moreover, the scavenging ability against the DPPH radical of samples increased over time. Noticeabily, the MNA2 sample revealed a DPPH radical‐scavenging ability of 82.6 ± 7% after 24 h, 1.2 times that of MNA1 and 3.4 times that of MNA0. It was attributed to the Arg released from APA particles, which have been reported as effective antioxidants with robust multiple ROS scavenging capabilities. Antioxidants can deactivate free radicals through two major mechanisms: hydrogen atom transfer and electron transfer.^[^ ^48^ ^]^ As depicted in Figure 4G, Arg molecules can act as antioxidants by donating electrons to free radicals, converting them into non‐radical structures. This process halts free radical chain reactions, preventing their accumulation and shielding organisms from oxidative damage caused by H_2_O_2_.^[^ ^49^ ^]^ Higher levels of Arg molecules enhance antioxidant capacity due to the increased availability of proton/electron donors. Additionally, the electron‐donating activity of functional groups is influenced by their electron‐cloud density; a higher density boosts this activity, thereby improving the ability to neutralize free radicals. Moreover, Arg is a precursor to NO, which has significant antioxidant properties. NO can inhibit the formation of free radicals via the formation of peroxynitrite (ONOO─), which can indirectly enhance the overall scavenging ability against radicals like DPPH.^[^ ^50^ ^]^ Due to the participation of Arg molecules in the APA particles, which are effective free radical scavengers, the resultant MNA patches exhibited promising antioxidant capacity. The wound site in patients with chronic wounds could produce excessive ROS because of its defense mechanisms. Low levels of ROS serve as essential mediators of intracellular signals for hemostasis, angiogenesis, and re‐epithelialization, which are critical for initiating wound healing. However, excessive ROS production or sustained oxidative stress can lead to local tissue damage.^[^ ^51^ ^]^ This induces cell death and triggers harmful processes such as necrosis, inflammation, and fibrotic scarring.^[^ ^52^ ^]^ In this regard, various strategies have been studied in the literature, including maintaining low levels of ROS or applying antioxidant materials. Our results demonstrated that the released Arg from the MNA patches maintained its strong antioxidant properties, showing the potential for eliminating excess ROS in skin wounds.

### 2.4 In Vitro Measurement of Intercellular ROS

**Figure 5:**
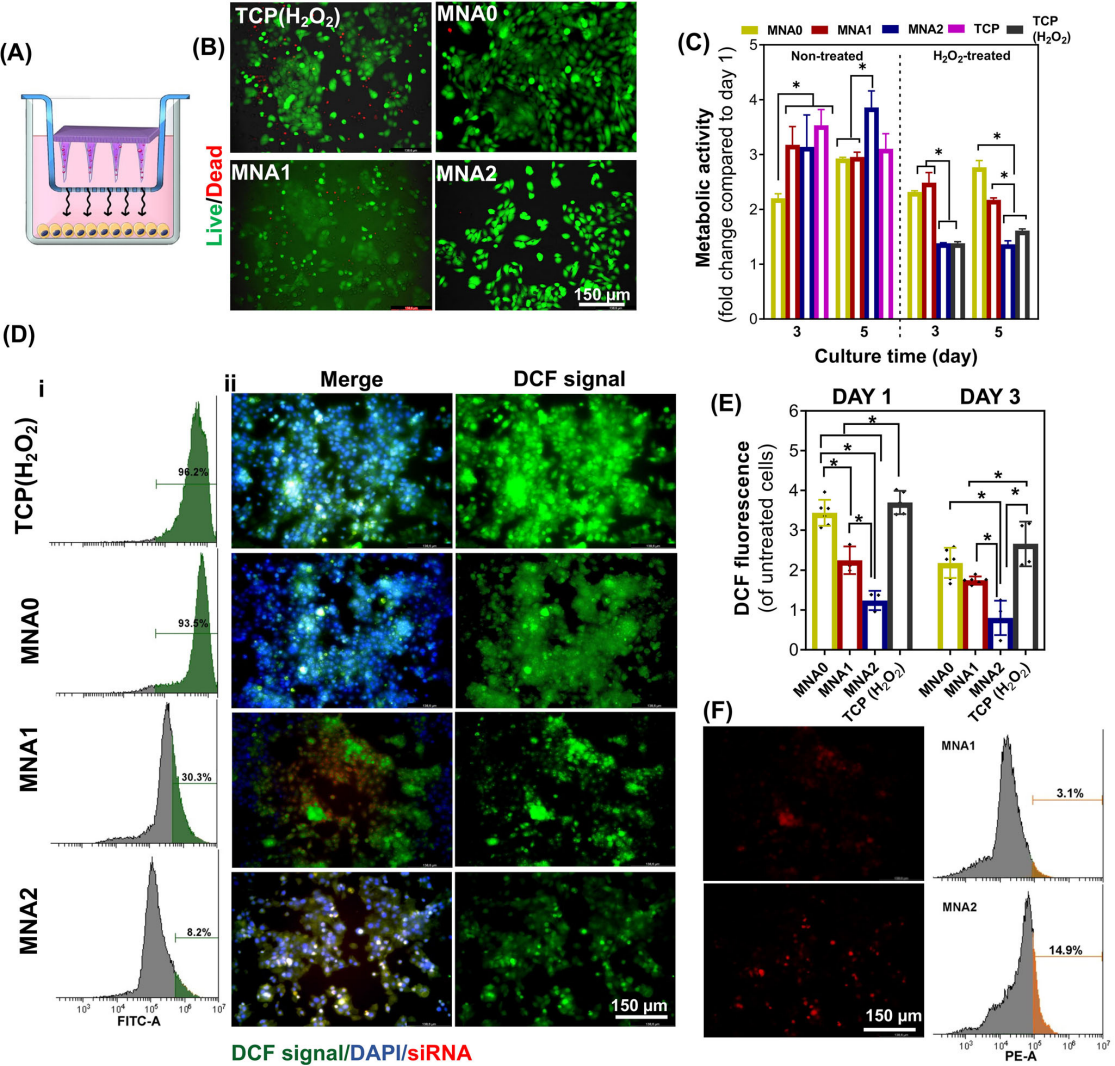
Improvement of the microenvironment for HaCaT cells through antioxidative effects of MNA patches: A) Illustration of the cell–MNA transwell model as a co‐cultured system. B) The representative fluorescent images of live/dead staining after contact with MNA patches and H_2_O_2_ treatment after 3 days of culture (cells were stained with calcein‐AM (green) and EthD‐1(red)). C) The effect of MNA patches on the metabolic activity of cells, before and after H_2_O_2_ treatment, was assessed by Alamarblue assay. D) The detection of intracellular ROS of HaCaT cells, after treatment with various MNA patches and incubation in H_2_O_2_: i) flow cytometry (The green color in the flow cytometry stands for intracellular ROS levels) and ii) fluorescence microscopy images of cells, following 3 days of culture. Red: siRNA‐labeled APA particles; blue: Nuclei; green: DCFH‐DA signal. E) Quantified DCFH‐DA signals after 1‐ and 3‐day incubation with H_2_O_2_ and treatment with various MNA patches (*P < 0.05). F) Intracellular uptake of Rhodamine B‐labeled APA particles after 3 days of incubation assessed via fluorescent microscopy and flow cytometry, compared to the control (untreated cells, TCP). The orange color in the flow cytometry stands for the portion of cells that uptake rhodamine B‐labeled APA particles.

During wound healing, an enhanced level of ROS is crucial for the early elimination of pathogens. Nevertheless, inflammatory responses and hyperglycemic conditions can lead to excessive ROS production in wounds, resulting in oxidative damage to cellular components and contributing to the development of chronic wounds. In this study, we validated the advantages of MNA patches regarding the prolonged siRNA release and antioxidant properties, subsequently investigating their efficiency in reducing ROS levels in vitro. Such properties could modulate the unfavorable wound microenvironment, supporting cell functions and enhancing tissue regeneration in chronic wounds.^[^ ^53^ ^]^ To demonstrate the inherent ROS scavenging ability of MNA patches, we explored their antioxidative properties in vitro using keratinocyte HaCaT cells and J774A.1 macrophages subjected to oxidative stress, induced by adding 3 mm H_2_O_2_ for HaCaT cells and 1 µg mL^−1^ LPS for J774A.1 macrophages. A transwell was applied to co‐culture MNA patches with HaCaT cells and J774A.1 macrophages (**Figure** **5**A). First, we assessed the viability and proliferation of HaCaT cells following treatment with MNA patches to evaluate the safety of particles released from the patches following H_2_O_2_ treatment. Given that HaCaT cells are essential in the wound healing process, we measured their viability after co‐culture with MNA groups, noting that it remained above 90% after 3 days of incubation without H_2_O_2_ (Figure 5B; Figure S6, Supporting Information). After treating the cells with H_2_O_2_ for 3 days, most of the cells were still alive; however, the viability of HaCaT cells slightly decreased when they were oxidatively stressed by H_2_O_2_ (Figure S6, Supporting Information). Specifically, when cells were exposed to H_2_O_2_, however, treated with MNA0, MNA1, and MNA2 patches, their viability was calculated at ≈76 ± 3%, 83 ± 2%, and 80 ± 2%, respectively, which were insignificant to each other.

Next, the proliferation of HaCaT was measured over 5 days of culture, focusing on changes in metabolic activity of cells, with and without treatment with MNA patches combined with H_2_O_2_‐induced oxidative stress. We observed an increase in the cell numbers after treatment with both MNA patches loaded with APA particles compared to pure MNA (Figure 5C). Furthermore, we noticed that after 5 days of culture, compared to the TCP group, the proliferative activity of cells in contact with the MNA0, MNA1, and MNA2 groups increased, confirming their cytocompatibility. Interestingly, the slower proliferation rate observed in HaCaT cells treated with MNA0 patches may be attributed to dissolved HA from the MNA, potentially acting as a barrier to cell proliferation and migration in the in vitro environment. On the contrary, after oxidative stress induced by H_2_O_2_, the proliferation rate of HaCaT cells significantly decreased, which correlated with the MNA patch compositions. The decreased proliferative activity of cells treated with MNA2 and the control sample exposed to H_2_O_2_ showed significantly lower values (*P* < 0.05), possibly due to the enhanced level of ROS generation in the absence of protective factors.

To assess ROS levels, we employed a ROS staining assay using DCFH‐DA (in green)^[^ ^54^ ^]^ and subsequently imaged the cells via fluorescence microscopy. Following its diffusion across the cell membrane, DCFH‐DA undergoes deacetylation by intracellular esterases and is then oxidized by the intracellular ROS into a highly fluorescent compound known as 2^′^,7^′^‐dichlorofluorescein (DCF), which can be quantified using spectroscopy.^[^ ^55^ ^]^ The green fluorescence signal of DCFH‐DA was analyzed using flow cytometry (Figure 5D‐i, after 3 days), fluorescence microscopy (Figure 5D‐ii; Figure S7A, Supporting Information, after 1 and 3 days), and spectroscopy (Figure 5E). Given the obtained results, an intense green fluorescence (DCF) was exhibited for HaCaT cells incubated with 3 mm H_2_O_2_ (TCP (H_2_O_2_)), signifying the elevated levels of the produced ROS, while only a small fluorescence signal was detected in the healthy control group. Our results demonstrated that the intracellular ROS levels of HaCaT cells treated with MNAs and then stimulated with H_2_O_2_ significantly decreased and were comparable to those in the control groups, ascribing to the decreasing oxidative stress and cellular injury.

The intracellular particles released from MNA patches were explored using the rhodamine B‐label. Fluorescence imaging results and flow cytometry analysis (Figure 5F; Figure S7B, Supporting Information) showed that particles could be detected intracellularly in contact with both MNA1 and MNA2 patches, and the intracellular fluorescence intensity of red color gradually increased from MNA1 to MNA2. Given that the efficiency of naked siRNA transfection is directly related to its uptake, we hypothesize that siRNA molecules likely diffuse laterally, leading to effective distribution within the needle insertion area. Additionally, the increased transfection efficiency was observed with MNA2. Furthermore, as shown in Figure 5E, the density of DCF fluorescence diminished with higher concentrations of APA. These findings suggested that adding APA can significantly inhibit the production of intracellular ROS. Together with the previous results, we could demonstrate that the uptake of particles by HaCaT cells helps remove excess ROS and transforms H_2_O_2_ into H_2_O and O_2_, thereby decreasing the formation of ⋅OH radicals. This process may alleviate oxidative stress and cellular damage, potentially aiding in reducing inflammation and enhancing tissue regeneration.^[^ ^56^ ^]^

**Figure 6:**
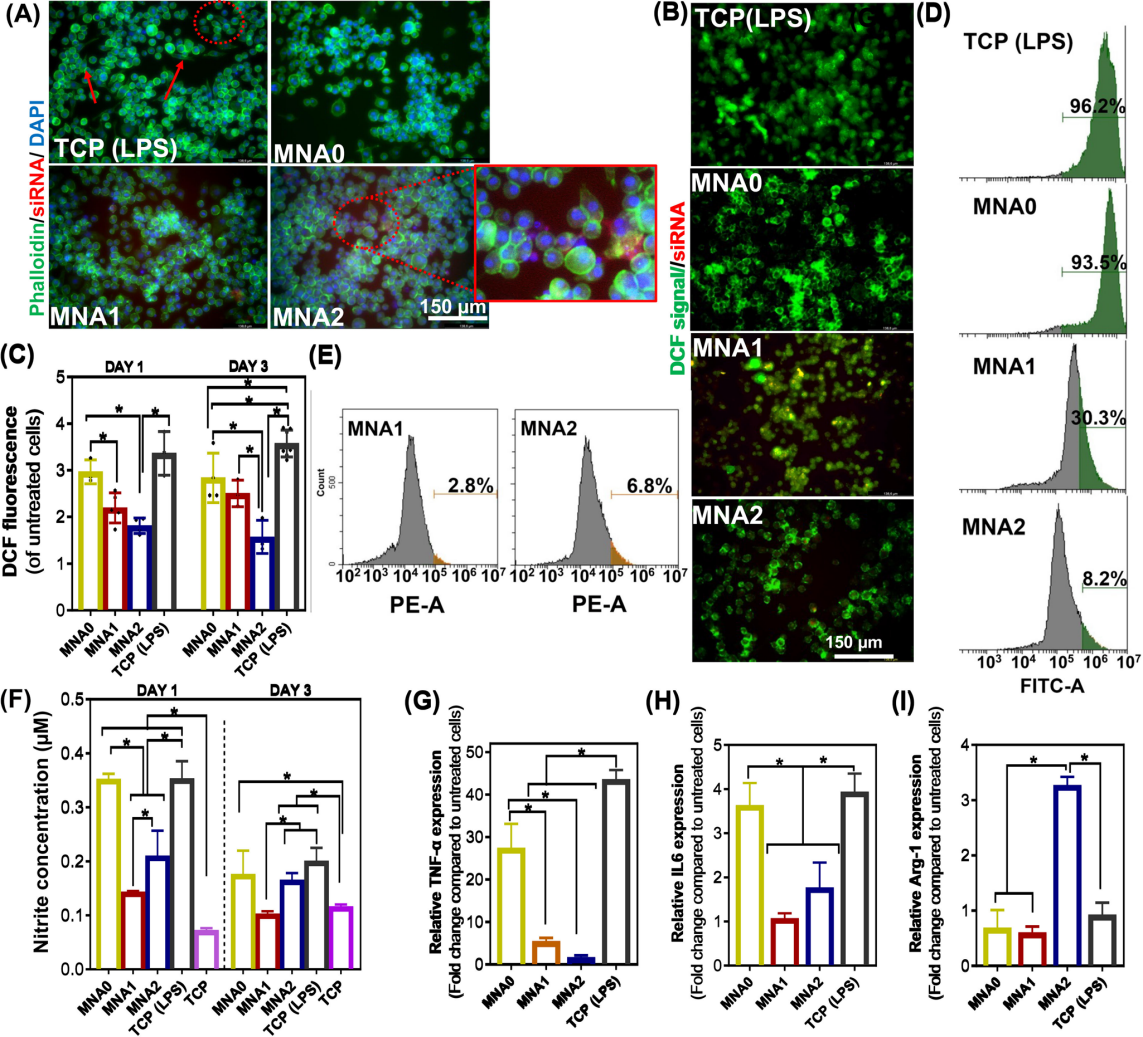
Immunomodulatory properties of MNA patches in contact with J774A.1 cells: A) Representative fluorescent image of macrophages following LPS stimulation and co‐culture with MNA patches and control (TCP (LPS)) after 3 days (Red: siRNA‐laden APA particles; blue: nuclei; green: F‐actin). Intracellular uptake of Rhodamine B‐labeled APA particles was identified in red color dots (High‐resolution image shows the typical uptake of particles). The spindle‐like M2 cells could also be identified (red arrows and circles show the spindle‐like cells). The detection of intracellular ROS of macrophages, following LPS stimulation and incubation with various MNA patches: B) fluorescence microscopic images of cells (Red: siRNA‐laden APA particles; blue: nuclei; green: DCFH‐DA signals), C) quantified DCFH‐DA signals, and D) flow cytometry analysis. The green color in the flow cytometry stands for intracellular ROS levels. E) Macrophages uptake Rhodamine B‐labeled APA particles released from MNA1 and MNA2 patches, tested via flow cytometry. The orange color in the flow cytometry stands for the portion of cells that uptake Rhodamine B‐labeled APA particles. F) The NO production in LPS‐pretreated cells in contact with various MNA patches after 1 and 3 days of incubation. In vitro macrophage repolarization assessed by quantitative polymerase chain reaction (qPCR) analysis G) TNF‐α and H) IL6 relative gene expression levels as M1 markers and I) Arg‐1 relative gene expression levels as M2 markers, conducted 1‐day post‐incubation. The gene expression levels were normalized to those of untreated cells. GAPDH was used as a housekeeping gene. Data are represented as mean (*n* = 3) ± SD (*P < 0.05).

To replicate the inflammation environment, J774A.1 macrophages were transfected with lipopolysaccharide (LPS) for 24 h and subsequently exposed to MNA patches. The ROS production was assessed after 1 and 3 days of culture, where it revealed that most of the cells (green‐stained cells) were alive after incubation with LPS and MNA treatment (Figure S8A,B, Supporting Information). The proliferation of macrophages treated with patches was measured using the AlamarBlue assay after 3 and 5 days of culture (Figure S8C, Supporting Information). Two types of controls were considered to compare the proliferation: cells cultured in well plate (TCP) as well as cells cultured in well plate and treated with LPS (TCP (LPS)). Compared with TCP, after LPS treatment in all groups, there was a noticeable decrease in the proliferation of macrophages. However, compared to day 1, cells proliferated with increasing culture time. Moreover, compared with TCP (LPS), there was an obvious growth trend of macrophage proliferation in cells treated specifically by MNA1 after 5 days of culture, indicating that these MNAs possessed good biocompatibility. This can be attributed to the metabolized arginine in the structure of APA particles. It is known that induced or overexpressed arginase promotes arginine metabolism, producing polyamines that contribute to some extent to macrophage proliferation.^[^ ^57^ ^]^ The morphology of most cells in TCP(LPS), after 5 days of culture, was spindle shape (**Figure** **6**A), while most of the cells, after co‐culture with MNA1 and MNA2 samples, turned to round shape, confirming that they may be modulated to the M2 phase. Furthermore, the improved cell proliferation after LPS pre‐treatment might be related to the ability of MNA1 and MNA2 to scavenge ROS. We determined the intracellular ROS levels using DCFH‐DA as a fluorescent probe and assessed the green fluorescence intensity using flow cytometry and fluorescence imaging. The fluorescence images presented in Figure 6B and the quantified DCF fluorescence signals in Figure 6C showed that J774A.1 cells in the control (LPS) group displayed an intense fluorescence, suggesting that inflammatory mediators stimulated ROS production.

Flow cytometry analysis further showed strong fluorescence signals for control TCP (LPS), indicating oxidative stress and stimulation of cells (Figure 6D). Moreover, the representative images demonstrated that applying APA particles, especially the MNA2 patch, led to a significant ROS reduction. Furthermore, Figure 6B,C showed that DCF fluorescence densities measured for cells treated by the MNA1 and MNA2 were markedly lower than TCP (LPS), suggesting that the incorporation of APA particles provided effective protection against LPS. The results in Figure 6C showed that MNA1 (2.7 level of untreated cells) and MNA2 (1.6 level of untreated cells) patches reduced the production of ROS by 28% and 58%, respectively, compared to TCP (LPS) (3.8 level of untreated cells), confirming the superiority of MNA2 in reducing ROS levels. It has been reported that the internalization of certain particles can take place specifically within the mitochondria to manage ROS levels.^[^ ^58^ ^]^ Consequently, we examined the effects of siRNA‐APA particle microtargeting on mitochondrial integrity and functionality. The fluorescence images in Figure 6B and flow cytometry analysis in Figure 6E show that the red fluorescence intensity of cells treated with polyplexes increased significantly with the increase in APA particle concentration in the MNA2 sample. This confirmed the uptake of particles by J774A.1 cells and the subsequent release of Arg from APA particles, which can stimulate ROS scavengers. In the context of ROS generation, Arg can activate antioxidant enzymes such as superoxide dismutase (SOD) and catalase. These enzymes help neutralize ROS by converting superoxide anions and hydrogen peroxide into less reactive species (peroxynitrite (ONOO^−^)), thereby reducing oxidative stress.^[^ ^59^ ^]^

Furthermore, the endogenous NO concentration released from J774A.1 cells was determined after 1 and 3 days of culture to evaluate the effect of Arg released from particles‐laden MNA patches on the ROS scavengers. A strong NO production was detected in cells seeded on the TCP (LPS) and MNA0 (*P *< 0.05) compared to the control (TCP), MNA1, and MNA2 patches after stimulation with LPS and pre‐treatment with MNAs for 1 and 3 days (Figure 6F). This could be attributed to the specific function of Arg in the Arg‐NO pathway. The Arg‐NO pathway plays a vital role in modulating oxidative stress and inflammation in the wound environment.^[^ ^60^ ^]^ Arg is released from the APA particles after internalization by J774A.1 macrophages. This release can be facilitated by cellular uptake mechanisms, such as endocytosis or micropinocytosis, described in our previous study.^[^ ^19^ ^]^ The release of Arg from APA particles led to the production of NO by nitric oxide synthases (NOS), which could activate various antioxidant enzymes, enhancing the cell's ability to scavenge ROS. For example, under inflammatory conditions, activated macrophages produce superoxide anions (O_2_^−^) through the activity of the enzyme NADPH oxidase. NO can enhance the expression of superoxide dismutase (SOD), which catalyzes the dismutation of O_2_^−^ into H_2_O_2_ and molecular oxygen (O_2_). NO may also stimulate the activity of catalase, which converts H_2_O_2_ into water and oxygen, thereby reducing oxidative stress. NO can also rapidly react with O_2_^−^ to form peroxynitrite (ONOO^−^), a potent reactive nitrogen species. ONOO^−^ can further decompose to form other reactive species, including hydroxyl and nitrogen dioxide radicals, contributing to oxidative and nitrosative stress. The reaction is highly dynamic and can occur rapidly, often within microseconds to milliseconds. In addition, our results showed that the MNA2 patch generated higher NO release than MNA1. Noticeably, after 3 days of culture, nitrate concentration released from cells in contact with MNA2 was 1.25 times greater than when in contact with MNA1. Thus, even though cells treated with MNA1 released a significant amount of NO, the antioxidant properties of APA particles could inhibit NO production in those cells. While NO and peroxynitrite have important signaling roles, excessive production can lead to cellular damage, prolong inflammation, and impair healing.^[^ ^61^ ^]^ Cell viability and proliferation results in Figure S8 (Supporting Information) and Figure 6 confirmed that the large quantity of ONOO^−^, which may be generated in contact with MNA2, can extend the healing period by prolonging the inflammatory phase. Therefore, the dynamic interaction between NO and superoxide anions is crucial for maintaining redox balance and influencing cellular responses during inflammation and wound healing.

### 2.5 In Vitro Mediating of M1 Macrophage Polarization

Pro‐inflammatory macrophages and cytokines are recognized for triggering host defense and enhancing phagocytosis, which are crucial roles in the early response to injury. However, the sustained presence of pro‐inflammatory macrophages can lead to chronic inflammation, which may impede the wound healing process. Hence, controlling the polarization of M1 macrophages in the early stages of wound healing is critical.^[^ ^62^ ^]^ To investigate the anti‐inflammatory effects of MNA patches, an LPS‐induced J774A.1 macrophage inflammatory model was employed. qPCR was utilized to analyze the secretion profile of macrophages. Given the data presented in Figure 6G–I, LPS‐treated macrophages exhibited a notable increase in the population of inducible TNF‐α and IL6‐positive M1 macrophages, with levels significantly reduced in the MNA2 sample. The MNA0 patch, however, had no obvious effect on the M1 macrophage marker expression. Notably, the TNF‐α and IL6 levels of M1‐positive cells were considerably lower in the MNA1 and MNA2 samples compared to the control group (TCP (LPS)), indicating the effective ROS scavenging effects of these two patches in suppressing macrophage M1‐polarization.

Further assessing the anti‐inflammatory properties of MNA patches, the anti‐inflammatory gene (Arg‐1) expression levels were measured using qPCR analysis. In the MNA0 group, there was a negligible effect on M1 polarization, while, significant increase in M2 polarization marker genes was observed in the presence of the APA‐loaded MNA groups (MNA1 and MNA2) (Figure 6I). Remarkably, after treatment using MNA1, the anti‐inflammatory M2 marker (Arg‐1) was significantly elevated (3.2 times) compared to TCP (LPS). The effects on macrophage polarization in the MNA0 were less pronounced than those observed in MNA1 and MNA2. The observed reduction in gene expression of M1 polarization markers (TNF‐α and IL6) and the increase in M2 polarization markers in the MNA1 and MNA2 groups confirmed the role of siRNA‐laden APA particles released from MNA patches in actively regulating macrophage polarization. Furthermore, the potential anti‐inflammatory activity of Arg released from APA particles, along with reduction of oxidative stress through ROS scavenging, contributes significantly to the regulation of macrophage polarization and the knockdown of Flii expression, makes APA‐laden MNA patches a great candidate for reducing the immune stress of the body during wound healing, providing a better basis for skin wound healing.

### 2.6 In Vitro Wound Healing Progress

**Figure 7:**
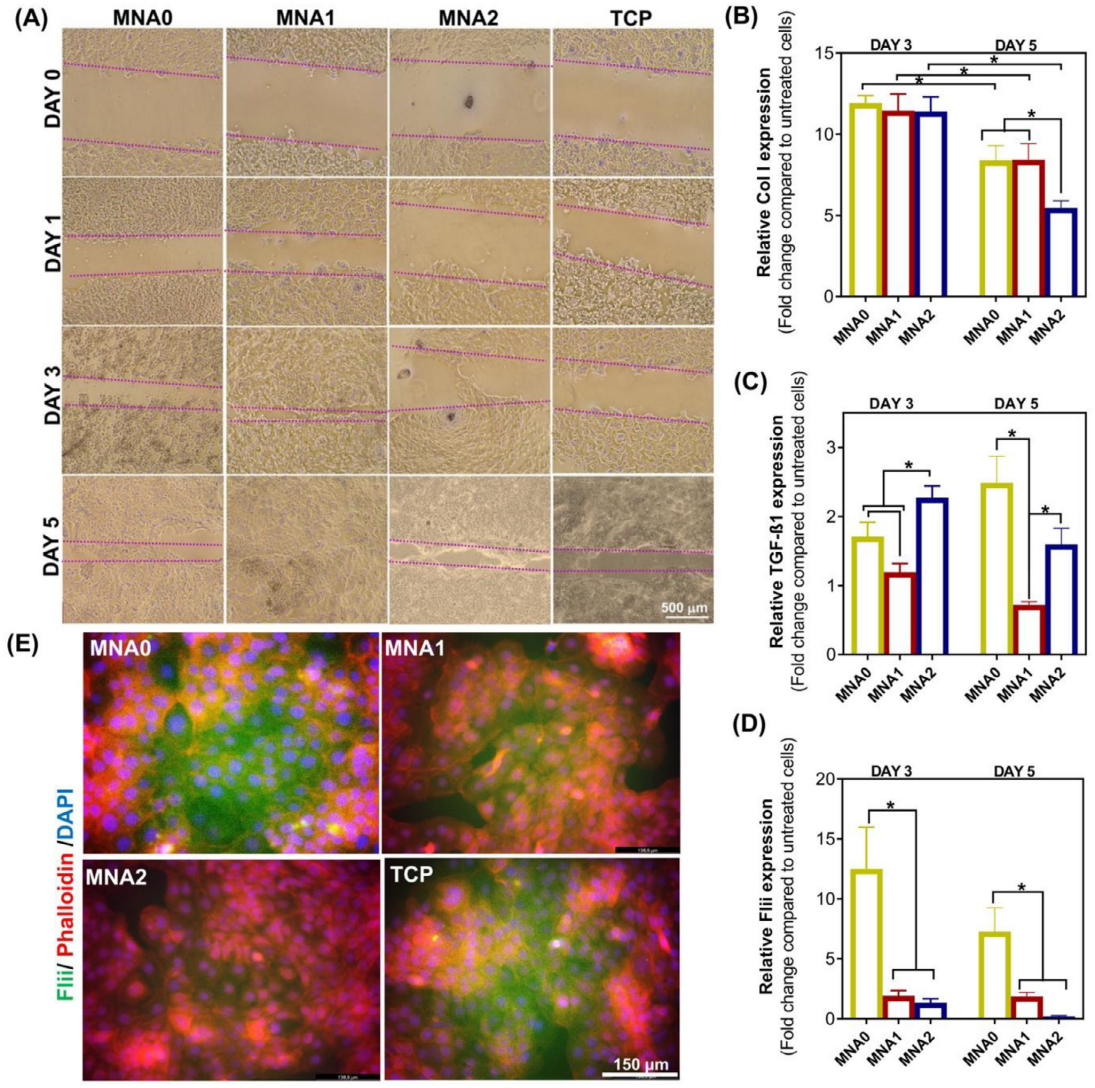
In vitro wound healing mediated by MNA patches: A scratch assay to evaluate wound healing ability by measuring HaCaT cell migration at 0, 1, 3, and 5 days post‐scratch; A) representative bright‐field images displaying the extent of scratch closure after treatment with MNA patches and control (TCP: untreated cells) at different time of culture (objective 5×). B) Col I and C) TGF‐β1 expression levels were analyzed using qPCR to assess wound healing‐related factors in HaCaT cells after 3 and 5 days of treatment with various MNA patches. D) Relative Flii expression was conducted after 3 and 5 days of incubation. The gene expression levels were normalized to untreated cells without any treatment, with CypA as the housekeeping gene. Results are presented as the mean (*n* = 3) ± SD with statistical significance indicated (*P *< 0.05). E) Fluorescent microscope images depicting the transfection of Flii Rhodamine‐siRNA using MNA patches, compared to untreated cells (TCP), in HaCaT cells after 5 days of incubation. The red, blue, and green colors represent F‐actin, cell nuclei, and Flii, respectively.

Extreme scarring poses a significant issue for skin repair, leading to severe functional and health difficulties for patients.^[^ ^63^ ^]^ To gain insight into the function of MNA patches in HaCaT cell migration and proliferation, we conducted a scratch wound assay with a micropipette tip. We used a transwell chamber and captured the images of the scratched cells at 0, 1, 3, and 5 days, where higher migration of HaCaT cells was detected after treatment with MNA patches, dependent on the compositions (**Figure** **7**A). There is a critical effect of siRNA release that interferes with the wound‐healing effect at these concentration ranges. We discovered that treatment with MNA1 significantly induced cell migration compared to that with MNA2 and MNA0 (Figure S9, Supporting Information). Notably, there was a clear distinction in the healing area between the MNA1 group and both MNA0 and control groups at all time points, while this difference was not significant in the case of MNA2 and controls. Noticeably, after 3 days of incubation, the scratch closure in the MNA1 group achieved ≈84%, while the closure area for other groups was less than 60%. It was estimated to be ≈35% in the case of MNA0.

Taken together, MNA1 demonstrated not only excellent biocompatibility but also a positive impact on the proliferation and migration of HaCaT cells, suggesting its potential to enhance wound healing. To further investigate the role of MNAs in wound healing and in vitro re‐epithelialization, we measured the expression of collagen type I (Col I) and TGF‐ß1 in HaCaT cells. The expression of Col I in cells treated with MNA1 and MNA2 patches was higher than the control (TCP), and improved up to 5 days (Figure 7B). Conversely, the expressions of TGF‐β1 genes were significantly reduced with the MNA1 treatment, compared to MNA0 and controls, indicating that MNA1 positively influenced the inhibition of scar formation and improved extracellular matrix remodeling (Figure 7C). It could be mentioned that TGF‐β1 is associated with fibrosis, and its suppression may lead to less scarring. According to the results, decreased Col I expression levels and increased TGF‐β1 levels after treatment with MNA2 compared to the MNA1 sample might be due to excessive NO release leading to delayed re‐epithelialization.

Improved cell migration and re‐epithelialization in contact with MNA1 compared to other groups might be due to the simultaneous role of the ROS scavenger ability and Flii siRNA release. To confirm this hypothesis, we evaluated the effect of Flii gene silencing by the MNA patch on HaCaT cells. As illustrated in Figure 7D,E, treatment with both MNA1 and MNA2 samples resulted in a decreased expression of Flii protein, with higher levels of green‐stained Flii correlating with increased cargo content (Figure 7E). Markedly, after 5 days of culture, cells treated with MNA1 and MNA2 patches exhibited a Flii gene expression change of 1.86 ± 0.12‐fold increase and 0.2 ± 0.06‐fold decrease, respectively, when normalized to non‐treated control cells (TCP). These findings indicate a significant downregulation of Flii gene expression in HaCaT cells treated with MNA1 and MNA2 patches, especially after 5 days, confirming the ability of these patches to achieve gene knockdown without inducing cytotoxicity (*P* < 0.05).

ROS plays a noticeable role in modulating Flii expression in keratinocytes. Acting as signaling molecules, ROS can activate transcription factors such as Nuclear factor‐κB (NF‐κB) and activated protein‐1 (AP‐1), which subsequently bind to the promoter regions of the Flii gene and enhance its expression. This protein is known to inhibit keratinocyte migration and proliferation, which are essential for effective wound healing.^[^ ^64^, ^65^ ^]^ Our results revealed that the silencing of the Flii gene has significant effects on the dynamic alterations in downstream signaling pathways and key molecules involved in keratinocyte behavior. Notably, Flii may influence the NF‐κB pathway, which is crucial for regulating inflammatory responses during wound healing.^[^ ^65^ ^]^ By modulating this pathway, Flii silencing could lead to altered expression of inflammatory cytokines such as TNF‐α and IL‐6, which are critical for keratinocyte migration and re‐epithelialization. Previous studies have indicated that changes in Flii expression can modify the inflammatory response, potentially resulting in prolonged inflammation and delayed healing.^[^ ^23^, ^65^, ^66^ ^]^ Furthermore, the mitogen‐activated protein kinase/extracellular signal‐regulated kinases pathway, known for its role in cell proliferation and migration, may also be affected,^[^ ^67^ ^]^ leading to reduced motility in keratinocytes, as confirmed by our findings on increased Flii levels correlating with suppressed migration. Furthermore, the enhanced levels of ROS associated with Flii downregulation can serve as signaling molecules that further promote these changes by activating pathways that impair cell shape and motility. Therefore, the interplay between Flii and these signaling pathways not only underscores the importance of Flii in wound healing but also elucidates the complex regulatory mechanisms underlying cellular responses to injury. Based on our results, the observed downregulation of Flii expression, combined with ROS scavenging capability and controlled release of Arg from MNA1, presents a favorable approach for promoting wound healing.

### 2.7 In Vivo Chronic Wound Healing Programmed Using MNA

**Figure 8:**
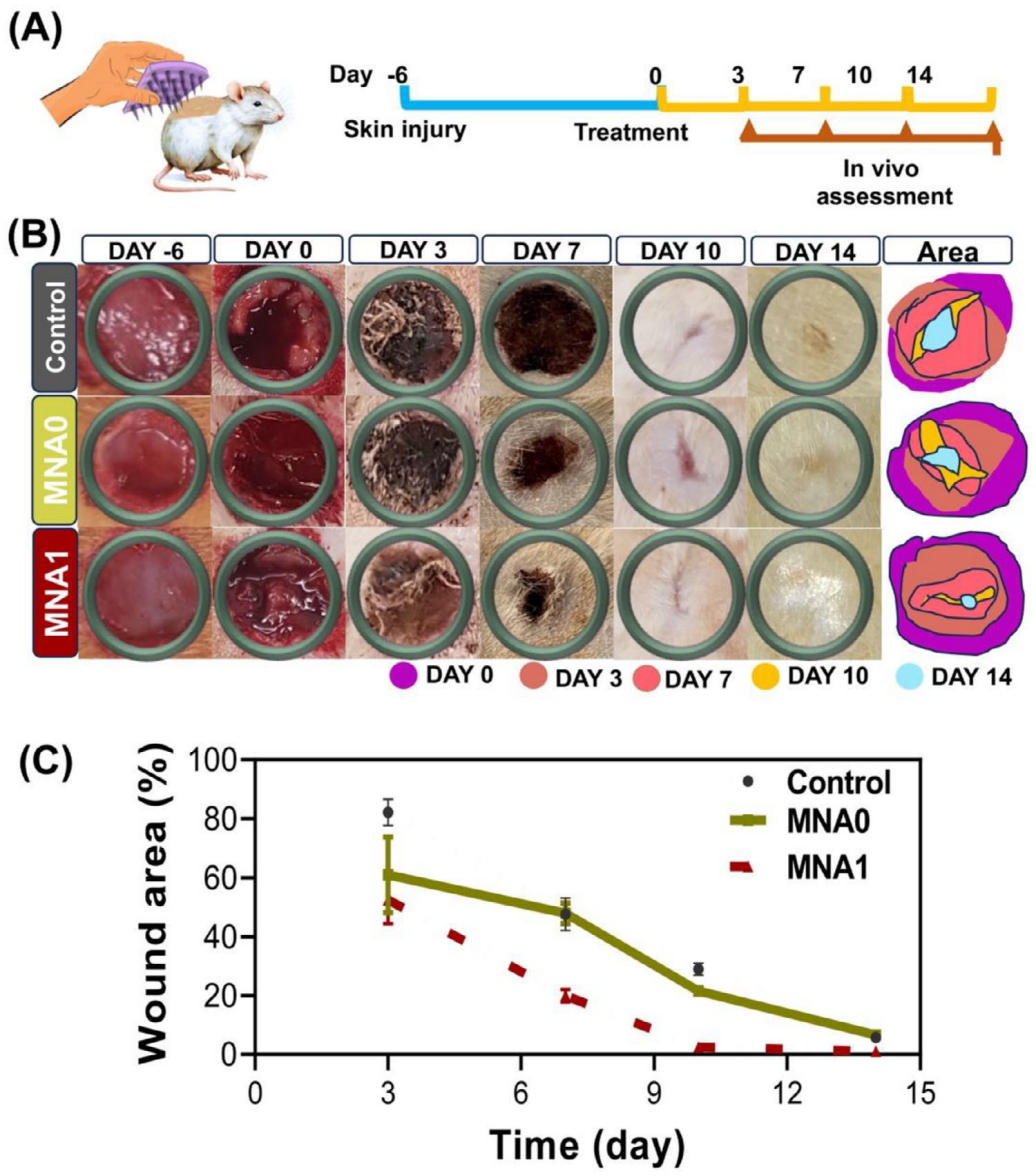
In vivo chronic wound healing mediated by MNA patches in rats: A) Schematic illustration of the MNA patches utilized for the rat wound model and the schedule of creating the inflammatory wound model and wound healing timeline. B) The representative optical images of the wound area and the schematic diagram of wound morphological changes at different times (Diverse colors signify various time points of the wound area map). C) The quantification of the wound closure rate (*n* = 3) of the chronic wounds at different healing times.

Encouraged by the significant capability of APA particles to control inflammation and stimulate cell proliferation and migration in vitro, we investigated the therapeutic effect of MNA patches to drive the healing process in a chronic wound model. We created a full‐thickness infected subcutaneous defect in healthy rat models by making 1 cm round wounds on their backs (**Figure** **8**A). In this section, the MNA1 patch, as the optimized sample, was studied under in vivo wound healing conditions in comparison with the MNA0 patch to study the efficacy of the siRNA‐laden APA particles encapsulated in the HaMA‐based MNA patch in chronic wounds. Accordingly, the rats were randomly assigned to three groups, including the control group (untreated wound), (2) MNA0, and (3) MNA1. Rats were subjected to a 1 × 1 cm excisional wound and received treatment with the MNA patches after 6 days. To evaluate the therapeutic effects in the different groups, the changes in the wound beds in each group were photographed on days 6 (before chronic wound formation), 0, 3, 7, 10, and 14 after the MNA implantation (Figure 8B), and the wound areas were determined using ImageJ software (Figure 8C). The wound closure rate indicated an upward trend in all the experimental groups, with the MNA1 group exhibiting the highest closure rate. While the control group showed no wound closure by the end of the first week, significant differences in the approximate rates of wound closure were observed between the two MNA1 and MNA0 groups after day 7. In animals treated by MNA1, the wound diameters decreased significantly from day 0 to day 3 (55%), the reduction size of the wound diameters slowed down from day 7 (80 ± 1%) to day 14 (98 ± 7%), and the wounds were gradually closed until day 14 (Figure 8C). On day 14, the wounds treated with MNA1 were almost completely healed with smooth and neonatal skin, while in the presence of MNA0, the wound did not completely close. The capability of APA particles encapsulated in MNA1 to promote wound healing in vivo was due to their strong ability to scavenge ROS and downregulate Flii expression. Previous studies suggested that Flii expression could negatively regulate macrophage TLR4 signaling, which reduced early inflammation via delaying the level and timing of TNF secretion, leading to impaired healing. And decreased the M2 macrophage phenotype, leading to impaired healing.^[^ ^66a^ ^]^ In another study, Flii‐neutralizing antibody was loaded into porous silicon nanoparticles to downregulate Flii overexpressed in a chronic wound.^[^ ^68^ ^]^ It was reported that the nanodelivery strategy could decrease the degradation of Flii‐neutralizing antibodies and effectively promote keratinocyte activation by reducing Flii expression, resulting in an accelerated wound healing rate in diabetic mice compared to the control group (≈18% faster). Moreover, the superior performance of HaMA in promoting wound healing and its notable moisturizing effects during degradation was also evident in the MNA1 group.

**Figure 9:**
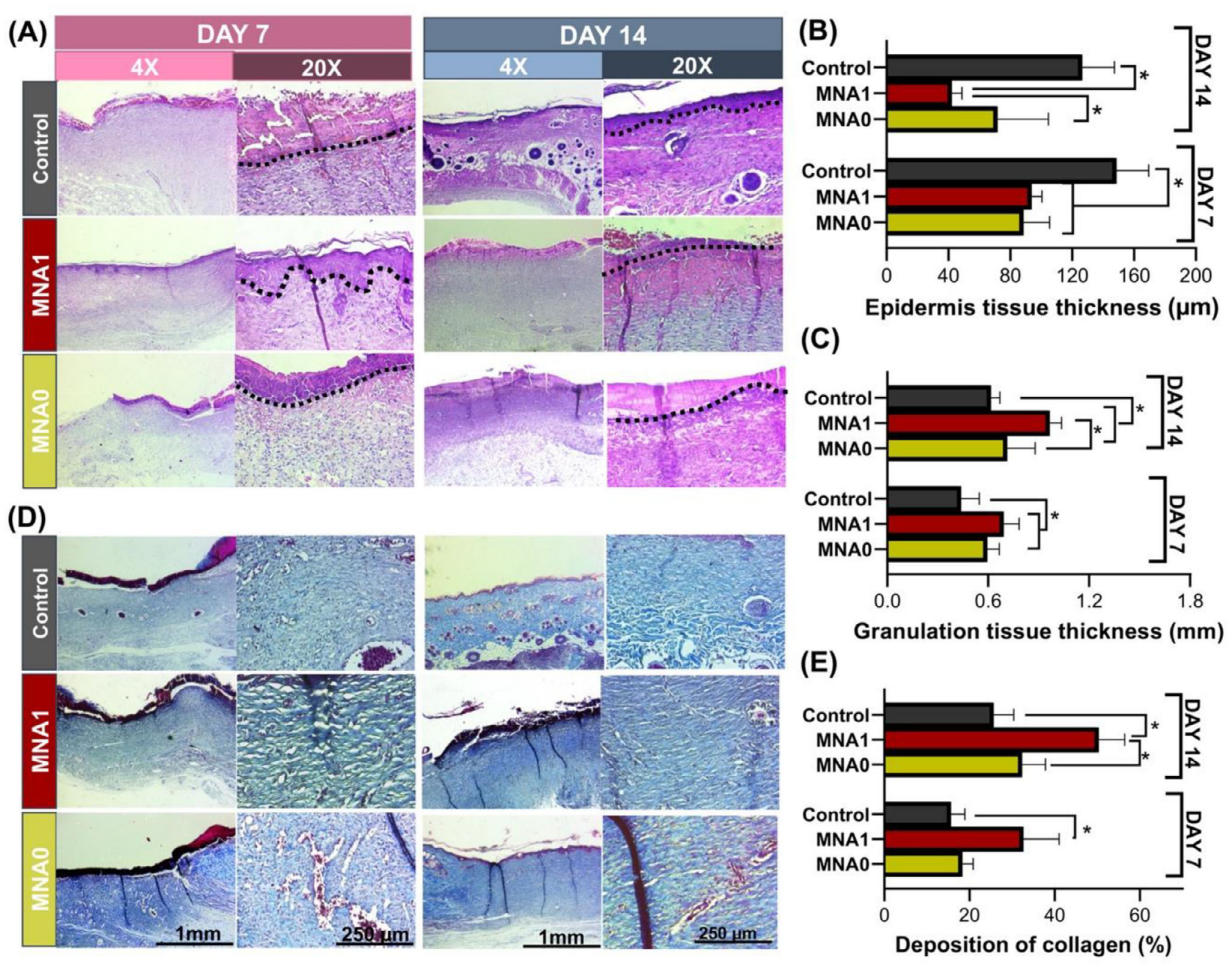
In vivo chronic wound healing mediated by MNA patches in rats: A) Representative images of H&E and their magnified images of wound sections in all groups after 7 and 14 days, respectively (scar area is identified above black dotted lines). The statistics of B) epidermis thickness (*n* = 3) and C) granulation tissue thickness for all groups on days 7 and 14 post‐wounding were determined using H&E staining. D) Representative images of Masson's trichrome staining images and their magnified images of wound sections in all groups after 7 and 14 days, respectively, and E) the deposited collagen (%) (*n* = 3) for all groups on days 7 and 14 post‐wounding calculated from Masson's trichrome stained images (* *P *< 0.05).

Typical markers of the wound healing process, including re‐epithelialization and the formation of granulation tissue, have been identified through histological analysis using hematoxylin‐eosin (H&E) and Masson Trichrome staining techniques (**Figure** **9**). Skin biopsies obtained for histological analysis were collected on days 7 and 14 following the treatment. In addition to the faster macroscopic closure, preliminary H&E histological analysis revealed the development of a volcano‐like shape during the first 7 days, mainly due to the newly formed tissue contracting inward from the edges, and re‐appearance of multiple hair follicles and sebaceous glands in the MNA1 group on day 14 (Figure  9A), whereas these features were not identified in the control and MNA0 groups. In the untreated group, the wound exhibited characteristic hypertrophic scar tissue, featuring a significantly thickened epidermis measuring 148 ± 20 µm at day 7, which subsequently decreased to 126 ± 25 µm after day 14 (Figure 9A,B). In contrast, the MNA1‐treated group revealed a wound structure with an epidermis that was flatter and threefold thinner (42 ± 7 µm) after 14 days, highlighting the substantial potential of MNA1 in preventing keloid formation and facilitating wound healing. Such significant restoration is a widely accepted hallmark of regenerative healing in the MNA1 group, rather than fibrotic healing.

The rapid repair of open wounds using MNA1 may be attributed to its positive interaction with the host's immune response, which accelerates tissue regeneration. According to our results, all groups exhibited differing levels of inflammatory cell infiltration. Significant infiltration of lymphocytes and red blood cells was observed at the wound sites treated with MNA0 and the control, indicating an inflammatory response. In contrast, the MNA1 patch treatment alleviated inflammation, as evidenced by the minimal presence of lymphocytes. This suggests that MNA1 substantially reduced inflammation within 7 days, promoting faster granulation tissue formation and subsequent epidermal remodeling. Granulation tissue is formed during the proliferation phase of defect healing, which is characterized by the proliferation of fibroblasts, keratinocytes, and endothelial cells, along with increased production of the ECM.^[^ ^69^ ^]^ Therefore, the thickness of the granulation tissue in all groups was measured (Figure 9C). After 7 days, the control group exhibited the thinnest granulation tissue formation (0.48 ± 0.1 mm). The MNA0 patch demonstrated a modest enhancement compared to the control group (0.59 ± 0.1 mm), while the MNA1 patch displayed the thickest granulation tissue of all the groups. On day 14, all groups demonstrated an increase in the thickness of granulation tissue. Notably, the granulation tissue in the MNA1 groups (0.35 ± 0.08 mm) was thicker than the control group. This group contained a great number of fibroblasts, blood vessels, and associated skin structures, such as hair follicles and sebaceous glands, compared to the other groups. Therefore, it created the fundamental structure of epithelium and dermis that closely resembled mature skin.

Furthermore, collagen deposition is essential for the functional repair of chronic wounds. The collagen deposited contributes to the strength of the newly formed tissue during the advanced stages of wound healing. Consequently, the amount of collagen deposition is one of the most important indicators for evaluating the effect of wound repair. Here, Masson's richrome staining was used to detect the deposition and appearance of new collagen on day 7^th^ or day 14^th^ after surgery (Figure 9D). The collagen content (%) of various groups is plotted in Figure 9E. Results showed that the control group revealed collagen bundle formation with enlarged thickness, uneven features, and a tightly packed and haphazard arrangement. Increased collagen thickness may enhance skin firmness but can also lead to greater skin stiffness and a higher risk of scar formation. Following MNA0 treatment, the epidermis of the wound was partially regenerated with high cellularity in the dermis, suggesting a potential for increased scarring. In contrast, in the MNA1 group, abundant regenerated collagen fibers were evident by day 7 post‐surgery. By day 14, all groups exhibited new collagen deposition and regenerated tissue formation; however, the MNA1 group had significantly more collagen deposition, with a regular orientation and even distribution, compared to the control and MNA0 groups. In the MNA1‐treated group, a significant amount of collagen fibers displayed a mature phenotype, identified by well‐structured alignment and thinner, more loosely organized bundles. This closely mimicked the collagen architecture of unwounded skin. These findings indicate that MNA1 can enhance chronic wound healing by facilitating fibroblast migration and proliferation, granulation tissue formation, and collagen deposition.

**Figure 10:**
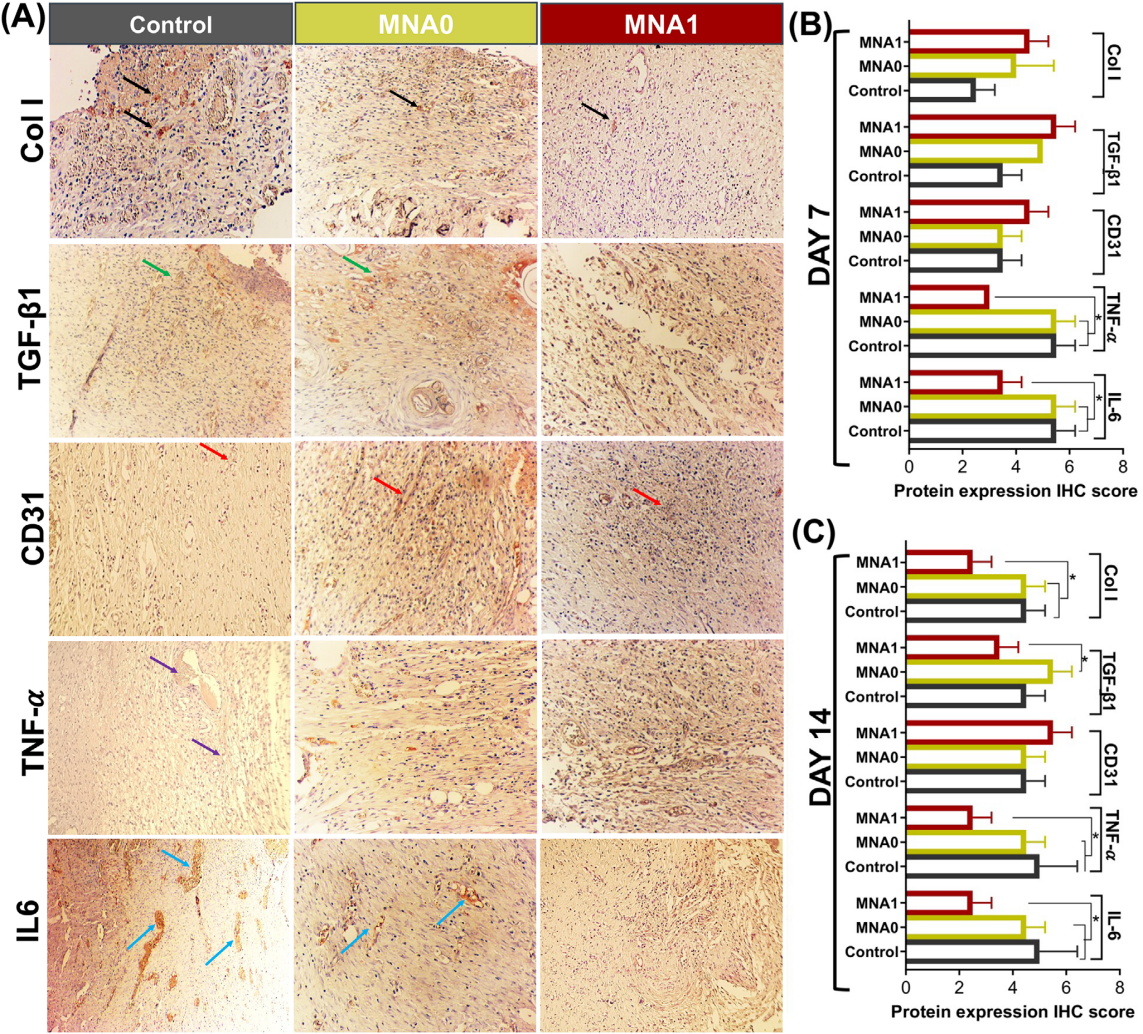
Therapeutic outcome of MNA patch for tissue remodeling of the inflammatory wound in a rat model. A) Immunohistochemistry (IHC) staining images of collagen type I (Col I, black arrows), TGF‐β1 (green arrows), CD31 (red arrows), IL6 (blue arrows), and TNF‐α (purple arrows) from the granulation tissues on day 14 of the wound healing process. The semiquantitative analysis in the expression levels of Col I, TGF‐β1, CD31, IL6, and TNF‐α after B) 7 and C) 14 days of the healing process (* *P *< 0.05). All histology images were captured at 100× magnification.

The Col I deposition in granulation tissues was measured using immunohistochemistry (IHC) staining assay in all the therapeutic groups on days 7 and 14 post‐repair (**Figure** **10**A; Figure S10, Supporting Information). All groups showed Col I deposition; however, in the MNA1 groups, a significantly lower deposited Col I was measured compared to the control and MNA0 groups (Figure 10B). No significant difference existed between the Col I deposition on MNA0 and controls at both measured time points. This might be attributed to the increase in the capacity of ROS scavenging and the reduced Flii gene expression that benefits from siRNA‐APA release in the MNA1 group. The results further confirm that our ROS‐scavenging wound dressing, which can downregulate Flii expression, could promote a wound‐healing process with minimal scarring. The expression of TGF‐β1 has been measured similarly (Figure 10A,B; Figure S10, Supporting Information) and showed an increase compared to the control group on day 7 in both MNA1 and MNA0‐treated groups. However, TGF‐β1 expression was downregulated in the MNA1 group by day 14 (Figure 10A,C). TGF‐β1 is released from platelets that degranulate at the injury position. It has been reported that the TGF‐β1 release will affect the inflammatory response, re‐epithelialization, extracellular matrix formation, and remodeling by initiating the primary signals that activate these processes. After an injury, in the wound microenvironment, TGF‐β1 is secreted by various cells, such as keratinocytes and macrophages, and its expression tends to increase during the inflammatory stage.^[^ ^70^ ^]^ On the contrary, lower levels of TGF‐β1 expression were identified in wounds that heal normally. Disruption in this expression pattern can lead to impaired wound healing, such as the formation of hypertrophic scars.^[^ ^71^ ^]^ It is reported that applying TGF‐β1 externally at particular stages of wound healing could result in effective treatments for patients with chronic wounds.^[^ ^72^ ^]^ Based on existing literature on TGF‐β1,^[^ ^73^ ^]^ our findings suggest that MNA1 may positively influence in vivo wound healing.

Beyond collagen deposition, the local vasculature is essential for delivering oxygen, nutrients, and vital growth factors to the wound, which are necessary for facilitating local regeneration. Nevertheless, chronic wounds often exhibit insufficient angiogenesis, underscoring the critical role of neovascularization in tissue repair. To assess the vascularization potential of the MNA patches, we measured capillary density around the injury site using immunohistochemical staining for CD31, a marker of endothelial cells and angiogenesis.^[^ ^74^ ^]^ As shown in Figure 10 and Figure S10 (Supporting Information), the MNA1 group significantly enhanced the formation of new blood vessels, exhibiting strong positive staining for CD31, particularly 14 days post‐surgery. Figure 10A–C presents the areas marked by CD31 signals in the MNA1‐treated group, which were greater than those in the MNA0 and control groups. Therefore, it further supported sustained angiogenesis, which is critical for appendage survival and long‐term tissue remodeling. We proposed that the faster wound healing could be due to the immune‐regulatory properties of the APA particles in the MNA1 sample.

Although we acknowledge that the difference in wound closure rate between the MNA1 and control groups was modest, the potential for accelerated wound healing is supported by several additional findings. The histological analysis (H&E and Masson's trichrome staining) revealed that the MNA1‐treated wounds formed more organized collagen bundles and showed restored skin features, such as hair follicles and sebaceous glands, which are indicative of regenerative healing. Moreover, the reduced expression of pro‐fibrotic markers, such as TGF‐β1, supports the hypothesis that MNA1 treatment mitigates fibrotic remodeling. Therefore, our results collectively suggest that the MNA1 system promotes a regenerative, rather than fibrotic, healing outcome. Taken together, the combination of reduced dermal thickness, organized collagen architecture, and enhanced angiogenesis provides indirect yet compelling evidence that MNA1 promotes a regenerative rather than fibrotic healing trajectory.

Although an appropriate inflammatory response is favorable for the healing process of a skin wound, excessive inflammation may impede wound repair. Therefore, inflammation serves as an important indicator of the infection level and the initial stage of the healing process.^[^ ^75^ ^]^ IHC staining for IL6 and TNF‐α was performed to validate the inflammation in wound sites. The results exhibited a high expression of IL6 and TNF‐α in the control group and MNA0‐treated groups after 7 days, signifying a severe inflammatory response in the wound site (Figure 10B; Figure S9, Supporting Information). However, the secretion of TNF‐𝛼 and IL6 after 7 days was considerably downregulated in the MNA1 group compared to that in the others (Figure 10B; Figure S10, Supporting Information). Such a phenomenon pointed out that the release of APA particles from patches and their distribution at the wound bed exhibited exceptional performance in not only decreasing inflammation but also encouraging the regeneration of neonatal epithelial tissues. Based on our in vitro and in vivo findings regarding macrophage recruitment and M2 macrophage polarization (Figure 6 and Figure 10), we concluded that the release of the APA particles resulted in the enhanced M2 macrophage population and the formation of a collagen‐rich ECM on the wound area.

The ability to promote wound healing while preventing scar formation is vital for the clinical performance of wound healing materials directly affecting skin regeneration and aesthetic results. Our research introduces a novel strategy utilizing an MNA patch to treat non‐healing wounds, emphasizing the importance of controlled modulation of the repair process to achieve healing. Recent studies reported the excessive production of ROS in chronic diseases, which often exceeded the capacity of the body's natural antioxidant systems, and can lead to harmful effects such as fibrotic scarring, and inflammation.^[^ ^76^ ^]^ In recent years, therapies targeting ROS have shown promise in enhancing wound repair and regeneration process.^[^ ^77^ ^]^ However, the relationship between ROS scavenging capabilities and the prevention of fibrotic scarring has not been widely explored. Here, we developed an MNA patch based on HaMA loaded with siRNA‐APA particles. The MNA1 patch can directly scavenge ROS to keep a relatively low level of ROS in the wound bed, which may create a favorable microenvironment, enabling the improvement of the survival and proliferation of macrophages and keratinocytes. On the other hand, the MNA1 patch could increase the antioxidant capacity in both macrophages and HaCaT, leading to reduced intracellular oxidative stress. This decline in oxidative stress within J774A.1 macrophage helps mitigate inflammation. In vivo decrease in the epidermal layer and TGF‐β1 downregulation were also related to the ROS scavenging ability and reducing inflammation by the MNA1 patch, providing a promising microenvironment for other types of cells to facilitate their roles in healing wounds. In addition to the ROS scavenging ability of the MNA‐based sample, the downregulation of Flii gene expression is another crucial reason for overcoming scar formation and accelerating wound healing. Wound injuries have been associated with increased production of Flii. ^[^ ^65^ ^]^ It plays a critical role in the fibroproliferative process that leads to hypertrophic scarring. Genetic elevation of Flii in animal models has resulted in greater dermal thickening and increased pivotal factors associated with hypertrophic scarring, such as myofibroblasts, TGF‐β1, and scar collagen composition.^[^ ^78^ ^]^ Our results showed that the reduced expression of the Flii gene yielded a lower expression of profibrotic genes like Col I and TGF‐β1, which is crucial for preventing wound fibrosis. Consequently, the decrease in Flii gene expression contributed to a downstream reduction in collagen production and scar formation. Chen et al.^[^ ^79^ ^]^ reported that the Flii overexpressing wounds resulted in increased TGF‐β1 expression, while TGF‐β3 was increased in Flii‐deficient wounds. Moreover, knocking down Flii gene expression via siRNA resulted in lower levels of TGF‐β1 and Smad 3 and elevated inhibitory Smad 7, suggesting a potential mechanism by which Flii influences TGF‐β1 signaling. In summary, our findings indicated that strategies focused on reducing inflammation, downregulating Flii, and scavenging ROS production may serve as effective therapeutic approaches for various types of chronic wounds, particularly for non‐healing wounds like those seen in diabetes or epidermolysis bullosa acquisita (EBA) disease.

## 3 Conclusion

In this work, we designed a multifunctional ROS‐scavenging and immunomodulatory MNA patch to facilitate local delivery of Flii sRNA in a minimally invasive way for the treatment of chronic wounds. The MNA patches demonstrated remarkable mechanical integrity with a strength of 0.6–1.6 N on the needle, enabling them to penetrate the tissue barrier at the wound site. In vitro cell culture experiments indicated that siRNA‐loaded MNs could effectively enhance the proliferation and migration of HaCaT and J774A.1 macrophages in harsh microenvironments. siRNA‐laden APA particles released from MNA patches not only effectively scavenged intracellular ROS but also promoted M2 polarization while down‐regulating M1 polarization of macrophages. Furthermore, we utilized a rat model with full‐thickness skin wounds to assess the in vivo therapeutic effectiveness of the MNA patch. Compared to the control group, the siRNA‐laden‐MNA showed a faster wound closure rate, enhanced epithelialization, less Col I deposition, and TGF‐β1 expression, and relatively complete CD31‐positive microvasculature. These features illustrated the high potential of the application of siRNA‐APA‐laden MNA patches for wound healing and gradual delivery of Flii siRNA as wound dressings. However, despite having superior antioxidant and anti‐inflammatory properties, our MNA patch does not address real‐time wound monitoring. Moreover, although the antioxidant performance was strongly supported by in vitro assays and the upregulation of anti‐inflammatory gene expression, direct assessment of ROS levels in vivo, was not performed, confining the mechanistic insight into ROS dynamics at the wound area. According to the above limitations, future research could focus on integrating a real‐time biosensing module into the MNA platform to track inflammatory or fibrotic markers in situ, allowing for feedback‐controlled release of therapeutic agents. Furthermore, future in vivo studies will contain redox‐sensitive imaging to confirm the ROS scavenging activity of the MNA patch in wound tissue.

## 4 Experimental Section

### Materials

*N, N*‐dimethylethylenediamine (Sigma), 1,4‐butanediol diacrylate (Sigma), dichloromethane (DCM, Merck), L‐Arginine (Arg, ≥99%, Carl Roth), *N*‐hydroxysuccinimide (NHS, Sigma Aldrich), and 1‐(3‐Dimethylaminopropyl)‐3‐ethyl carbodiimide hydrochloride (EDC‐HCl, Cabosynth) were used to synthesis L‐Arginine functionalized poly(β‐amino ester) (AP). Sodium alginate (Carl Roth) and the rhodamine‐labeled Flii siRNA with sequences of GCUGGAACACUUGUCUGUGUU and CACAGACAAGUGUUCCAGCUU (Flii Rh‐siRNA, Qiagen) were used to synthesize siRNA‐laden APA polyplexes (siRNA‐APA particles). Sterile nuclease‐free diethylpyrocarbonate (DEPC) water (Invitrogen) was applied to synthesize APA particles. Hyaluronic acid sodium salt (HA, M_W_ 1–2 × 10^6^ Da, Carbosynth), methacrylic anhydride (MA, Sigma‐Aldrich), acetone (Merck), sodium hydroxide (NaOH, Labochem), and ethanol (Sigma‐Aldrich) were provided to synthesize HaMA. Dialysis tubes with a molecular weight cutoff 12–14 kDa were purchased from Thermo Fisher Scientific. Polydimethylsiloxane (PDMS) pyramidal micromolds with needle cavities of 300 µm were provided by Micropoint Technologies (Singapore). This master mold comprised 100 pyramidal needles arranged in a 10 × 10 grid, with a tip‐to‐tip distance of 500 µm between the needles. N‐vinyl caprolactam (VC, Sigma), Eosin Y (EY, Sigma), and triethanolamine (TEA, Sigma) were purchased to fabricate HaMA‐based MNA patches. Methylene blue (MB) and hyaluronidase (HAase) were provided by Sigma to study the microneedle penetration and degridation rate, respectively.

Two cell types of HaCaT (keratinocyte cells, passage number < 10) provided by DKFZ, CLS, Germany, and J774A.1 (macrophages from ascites, passage number less than 10, American Type Culture Collection (ATCC, Manassas, USA, TIB‐67) were tested in contact with MNA patches. For cell culture, Dulbecco's Modified Eagle's Medium (DMEM, BioSell), fetal bovine serum (FBS, BioSell), 10× Trypsin‐EDTA (Gibco), penicillin‐streptomycin (Pen/Strep, Gibco), Gentamycin‐Sulfate (Sigma‐Aldrich), GlutaMax (Thermo Fisher Scientific), 2‐ [4‐ (2‐hydroxyethyl)piperazin‐1‐yl]ethanesulfonic acid (HEPES, Thermo Fisher Scientific), Opti‐MEM reduced serum medium (Thermo Fisher Scientific, US), and Dulbecco's Phosphate Buffered Saline (DPBS, Sigma) were used. Cell evaluation behavior was assessed using paraformaldehyde (PFA, Carl Roth), Alexa Fluor 488 Phalloidin (Invitrogen), Phalloidin Tetramethylrhodamine B isothiocyanate (Rhodamine phalloidin, Sigma), 4′,6‐diamidino‐2‐phenylindole (DAPI, Sigma Aldrich), bovine serum albumin (BSA, Sigma Aldrich), Flii Polyclonal Antibody (Invitrogen) and Alexa Fluor 488 goat anti‐rabbit IgG SFX kit Thermofisher, A31627). In addition, Alamarblue Cell Viability Kit (Promega), Nitrite Assay Kit (Griess Reagent, Sigma Aldrich), Live/Dead Viability Kit (Calcein AM‐Ethidium Homodimer‐1 (EthD‐1), Invitrogen), lipopolysaccharide (LPS, Med Chem Express (MCE)) and 2′,7′‐Dichlorodihydro‐ fluorescein diacetate (DCFH‐DA, Med Chem Express USA) was applied for further cell behavior studies. For PCR assays, the RevertAid First Strand cDNA Synthesis Kit (Thermofisher) and innuMIX qPCR DSGreen Standard (Analytik Jena) were provided. The list of primers purchased from Bio‐Rad is also provided in Table S1 (Supporting Information). Hematoxylin and eosin staining (H&E) and Masson's trichrome stains were provided by Solarbio, G1340. Collagen type I (Col I, GTX26308, GeneTex, USA), Transforming growth factor (TGF)‐β1 (MAB240, Bio‐techne, USA), interleukin (IL)‐6 (orb10911, Biorbyt, UK), Platelet endothelial cell adhesion molecule (named as cluster of differentiation 31 (CD31, Clone JC/70A, Vitro SA, Spain), TNF‐α (Orb371962, Biorbyt, UK) antibodies and goat‐anti‐rabbit IgG secondary antibody (Zhongshan Biology Company, China) and goat‐anti‐mouse IgG secondary antibody (Zhongshan Biology Company, China) were used to assess in vivo studies.

### Synthesis of AP Polymer

Poly(β‐amino ester) (PAE) and Arg functionalized PAE (AP) polymers were synthesized according to the previous research.^[^ ^19^ ^]^ Briefly, PAE was synthesized via a Michael reaction between *N, N*‐dimethylethylenediamine and 1,4‐butanediol diacrylate at the diacrylate: diamine monomer molar ratio of 1.5:1 in DCM solution. Moreover, AP polymer was synthesized based on EDC/NHS chemistry in which Arg was linked to PAE polymer at a weight ratio of 1:3 using NHS and EDC‐HCl.

### Formation of siRNA‐Laden APA Polyplex

The siRNA‐laden APA polyplexes (siRNA‐APA particles) at an APA: siRNA weight ratio of 100:1 were formed via the incorporation of equal volumes of Flii siRNA solution (0.623 mg mL^−1^ in sterile nuclease‐free DEPC water) to AP, according to the previous study.^[^ ^19^ ^]^ The solutions were mixed by pipetting and vortexing for 15 s and then incubated at room temperature (RT) for 10 min. Then, an equal volume of 0.623 mg mL^−1^ alginate solution in sterile nuclease‐free DEPC water was added, mixed, and incubated for an additional 10 min. As‐prepared particles were centrifuged for 10 min at 10 000 g and kept at 4 °C for the following experiments.

### Synthesis of Hyaluronic Acid Methacrylate (HaMA)

HaMA was synthesized according to the procedure reported by Uribe‐Gomez et al.^[^ ^35^, ^80^ ^]^ Briefly, a 3 wt.% HA solution was prepared in 50 mL of deionized water. Following the addition of methacrylic anhydride (0.8 mL), the solution was mixed for 3 h while its pH was fixed to 8–9 using 1 m NaOH. The mixture was continuously stirred for 24 h, and acetone was added to precipitate a milky white substance, and rinsed with ethanol to remove the remaining methacrylic anhydride. The product was then dissolved in deionized water and dialyzed for 2 days at RT. Ultimately, HaMA was achieved by lyophilization for 48 h.

### Fabrication of HaMA‐Based MNA Patch

The flexible HaMA‐based MNA patches were fabricated by adapting the micro‐molding using a unified PDMS mold. First, a HaMA (20 mg mL^−1^) solution containing 0.75 wt.% TEA and 0.5 wt.% VC was prepared in 2 mL of DEPC water. Following the addition of 1 µL of a 0.5 mm Eosin Y solution, the siRNA‐laden APA particle suspension (0.623 mg mL^−1^)^[^ ^19^ ^]^ was dispersed in HaMA solution to achieve final particle concentrations of 5 wt.% and mixed for 20 min at RT. The concentration of TEA, EY, and VP was optimized according to the initial trial‐and‐error experiments and based on the previous studies.^[^ ^81^ ^]^ Pure HaMA solution was similarly prepared separately.

A two‐step procedure combining centrifugation and vacuum molding was utilized to create siRNA‐laden APA‐MNA patches. First, 150 µl of the HaMA‐based suspension was poured into the PDMS mold. The mold was subjected to a vacuum for 3 min, followed by centrifugation at 4000 rpm for 3 min at 5 °C (Centrifuge Heraeus Multifuge 3SR Plus, Thermo Fisher Scientific, Waltham, MA, USA) to ensure the mixture filled the mold. The second layer of HaMA was made after pouring 150 µL of the HaMA‐based suspension into the molds. It was centrifuged similarly and kept in the dark for 4 h. Next, a blank HaMA‐based solution was added to the surface to form the backing. The excessive HaMA was removed via centrifuging for another 5 min. The samples were preserved overnight at 50% relative humidity and room temperature in the dark for 8 h. Finally, the MNA patches were peeled off from the mold and exposed under green light (365 nm) for 20 min, where the pink MNA patches were crosslinked into a clear, soft, flexible yellow hydrogel. To investigate the role of siRNA‐laden APA particles on the HaMA‐based MNA properties, the composition of the HaMA‐based solution in the first two steps was varied. While pure HaMA was used for the first layer of MNA1 patches, a siRNA‐APA‐laden HaMA suspension was used for the second layer. In the MNA2 patch, both first layers were made of siRNA‐APA‐laden HaMA suspension. Pure HaMA made MNA0 as both the first and second layers were used as controls.

### Physicochemical Characterization

The functional groups of HA, HaMA, and MNA patches were identified using FTIR spectroscopy (Bruker Tensor 27, USA) with spectral data spacing of 4 cm^−1^ from 800 to 4000 cm^−1^. ^1^H NMR spectra of HA and HaMA were also investigated using an INOVA‐400 spectrometer at 400 MHz. Deuterium oxide (D_2_O, Cambridge Isotope Laboratories) was used as the solvent. The degree of methacrylation (DM%) was determined from the relative integrations of the methacrylate protons (vinyl protons) to HA protons by using the following Equation (1):^[^ ^82^ ^]^

(1) $$DM\%=\left\lfloor \frac{a+b/2}{c/3}\right\rfloor \times 100$$

where *a* and *b* represent two substituting methacrylate groups detected by ^1^H NMR peaks, and *c* denotes the HA backbone comprising 3 peaks from 3–4.2 ppm. The structural features of MNA patches were assessed through a stereomicroscope (Nikon SMZ25), a scanning electron microscope (SEM, Carl Zeiss Microscopy GmbH, and Apreo VS, Thermo Fisher Scientific, Germany), a fluorescence microscope (a Leica DMI 3000B), and confocal laser scanning microscopy (Leica DMI 8  equipped with lasers using LAS X software). The Z‐potential and hydrodynamic diameter of APA and siRNA‐APA particles were determined using dynamic light scattering (DLS, Litesizer 500, Anton Paar).

The mechanical characteristics of HaMA‐based MNA patches were evaluated using a mechanical testing machine (ElectroForce 3200, TA Instruments, DE, USA), following the previously outlined procedure.^[^ ^39^ ^]^ In summary, the MNA patches were mounted on the bottom plate of the tensile test machine with the needle tips oriented upward. An axial force was applied with a load cell of 2.5 N, acting perpendicularly to the MNA's axis at a consistent speed of 1 mm s^−1^. The process was conducted in triplicate. The testing machine recorded the force needed to displace the mount as a function of microneedle movement. By converting the linear part of the force–displacement curve, the compressive modulus was extracted as the stiffness index of the MNA patches. After completion of the experiment, MNAs were once more inspected through a stereomicroscope to detect any MNA geometric alterations created by the compression forces.

The swelling ability and degradation rate of MNA patches were investigated in PBS buffer (pH = 7.4) at 37 °C. MNA patches were weighted (*W*_1_) and immersed in 5 mL of PBS solution. During the 5 days of incubation, following the drying of the surface water with filter paper, the samples were weighed (*W*_2_). The swelling ratio of the MNA patches was determined according to Equation (2):

(2) $$Swelling\;ratio\;\left(\%\right)=\frac{W_{2}-W_{1}}{W_{1}}\times 100$$

To determine the degradation rate of MNA patches, the samples were freeze‐dried and weighed (*W*_1)_. Then, they were soaked in 5 mL of PBS solution containing 5 U mL^−1^ of HAase in a constant temperature shaker (37 °C, 70 rpm) for 14 days. The concentration of HAase was selected according to previous studies.^[^ ^83^ ^]^ At different time points, the patches were taken out, rinsed with ultra‐pure water, freeze‐dried, and accurately weighed (*W*_2_). Finally, the degradation rate was measured according to Equation (3):

(3) $$\mathrm{Weight}\;\mathrm{loss}\;\left(\%\right)=\frac{W_{1}-W_{2}}{W_{1}}\times 100$$

### In Vitro/Ex Vivo Penetration Experiments

The penetration of MNA patches containing rhodamine B‐labeled siRNA was investigated using agarose gel, which was used to mimic the dermis layer of the skin tissue.^[^ ^45^ ^]^ To prepare the agarose gel piece, a 1.5 wt.% agarose solution was heated in a microwave and poured into a 3 mm × 3 mm petri dish, forming a 2 mm agarose gel. Following administration for 1 min, the Rhodamine B‐labeled MNA patch was imaged using the fluorescent microscope to show the penetration depth of MNAs in the agarose layer. Moreover, 1 mg mL^−1^ of methylene blue (MB) was loaded in an MNA1 patch (as an example) to evaluate the visual release of MB after 30 min, 2 h, and 6 h. Furthermore, an *ex vivo* test was conducted to determine the ability of the MNA patches to penetrate the rat cadaver skin immediately after the euthanasia of the animal, following established methodologies.^[^ ^84^ ^]^ The animal procedure will be discussed in “In vivo wound healing evaluation” section. In brief, the MNA patches were pressed onto the skin using thumb pressure for 30 s and left in place for 1 h. Following removal, the sites where the MNA patches penetrated were harvested from the skin and fixed in 10% formalin for 48 h. The fixed skin was paraffin‐embedded and then sliced into 5‐micrometer‐thick sections using a cryotome (MC 4000 microtome cryostat, Histo‐Line Laboratories, Italy) and placed on glass slides. Finally, the slices were stained following the standard Masson's trichrome staining protocol. The samples were then examined using the full slide scanner (Zeiss AxioScan.z1, Japan).

### siRNA Release and Stability Evaluation

To investigate the amount of siRNA loaded, un‐crosslinked MNA patches were initially removed by scraping the needles into an Eppendorf tube and subsequently re‐dissolved with DEPC water. The concentration of siRNA encapsulated in MNA patches was then measured using a NanoDrop ND‐1000 UV–vis Spectrophotometer (Thermo Fisher Scientific) at 260 nm. The MNA0 patch, which did not contain siRNA, served as a control to account for any background absorbance attributed to HaMA and particles. Moreover, the siRNA release from MNA patches was determined in PBS (pH = 7.4) for 5 days at 37 °C. The quantity of the released siRNA was determined using a NanoDrop ND‐1000 UV–vis Spectrophotometer (Thermo Fisher Scientific) at a wavelength of 260 nm, and siRNA release (%) was calculated according to the following Equation (4):^[^ ^85^ ^]^

(4) $$Released\;siRNA\;\left(\%\right)=\left(\frac{OD\;of\;siRNA\;in\;PBS}{OD\;of\;initial\;content\;of\;siRNA}\right)\times 100$$

### 1,1‐Diphenyl‐2‐Picrylhydrazyl (DPPH) Free Radical Scavenging Assay

The ROS scavenging ability of MNA patches was evaluated by measuring their inhibitory effect on the 1,1‐diphenyl‐2‐picrylhydrazyl (DPPH) radicals.^[^ ^47^ ^]^ Briefly, 20 mg of MNA patch was mixed with 3 mL of 0.5 mm DPPH solution in ethanol. The mixture was stirred at a constant rate of 200 rpm for 5 min and then incubated for 60 min. Finally, the absorbance of the solutions was recorded at 517 nm using a UV–vis spectrophotometer, and the DPPH radical inhibition rate was calculated using Equation (5):

(5) $$DPPH\;radical\;\mathrm{scavenging}\;\mathrm{activity}\;\left(\%\right)=\frac{\left(A_{0}-A_{1}\right)}{A_{0}}\times 100$$

where *A*_0_ and *A*_1_ correspond to the absorbance of the DPPH solution before and after the reaction with MNA samples, respectively.

### In Vitro Cellular Experiments

J774A.1 macrophages and HaCaT human skin cell lines were applied to investigate cell‐MNA patch interactions. J774A.1 macrophages were cultured in DMEM supplemented with 10% FBS, 2% HEPES, 2% GlutaMax, 1% sodium pyruvate, and 1% Pen/Strep. Similarly, HaCaT cells were cultured in DMEM high glucose supplemented with 10% FBS and 1% antibiotic‐antimycotic at 37 °C and 5% CO_2_.

### Cell Viability and Proliferation Assay

The viability and proliferation of J774A.1 macrophage and HaCaT cells in contact with MNA patches were assessed using live/dead and Alamarblue assays, respectively. The J774A.1 macrophage and HaCaT cells, with initial densities of 2 × 10^4^ and 10^4 ^cells per well, respectively, were seeded in 24‐well plates and incubated in complete medium at 37 °C for 24 h. The samples were sterilized under UV light for 20 min and placed in Transwell inserts within each well. The culture medium was then replaced with 1 mL of Opti‐MEMTM I reduced serum medium. Untreated cells were used as the positive control (named tissue culture plate (TCP)).

To evaluate cell proliferation at the specific time points (1, 3, and 5 days), the Alamarblue solution diluted in the full medium at a 1:10 volume ratio was added to the cells. After 4 h incubation, the absorbance of the medium was determined at 560/590 nm (ex/em) using a plate reader (Mithras LB 940, Bertold, Bad Wildbach, Germany).

Furthermore, cell viability was further evaluated using a live/dead fluorescence assay based on the simultaneous application of two fluorescent dyes: calcein‐AM and ethidium homodimer‐1 (EthD‐1), following the manufacturer's instructions. Briefly, cells were incubated with 100 µL of staining solution containing 2 µm calcein AM and 4 µm EthD‐1 for 20 min at room temperature in the dark. After incubation, stained cells were visualized using a Leica DMI 3000B fluorescence microscope equipped with a 20× objective lens. Excitation/emission (ex/em) filter settings of 495/515 nm and 496/635 nm were used to detect calcein‐AM and EthD‐1 signals, respectively.

### In Vitro Antioxidation Assay

To determine the antioxidant properties of MNA patches, HaCaT cells were incubated with MNA patches in 24‐well plates for 5 days, following the procedure described in “*Cell viability and proliferation assay*”. The initial seeding density of J774A.1 macrophage and HaCaT cells were 2 × 10^4^ and 10^4 ^cells per well, respectively. After 1 and 3, days of incubation, the culture medium was replaced with DMEM containing 1 mm H_2_O_2_, and incubated at 37 °C for 4 h, according to the protocol established in previous studies.^[^ ^86^ ^]^ Cells treated only with H_2_O_2_ and untreated cells served as the negative (TCP (H_2_O_2_)) and positive (TCP) controls, respectively. After 1 and 3 days of culture, mitochondrial function in HaCaT cells was assessed by evaluating intracellular ROS. Cells were stained with dichlorofluorescein diacetate (DCFH‐DA, 10 µm in fresh culture medium) at 37 °C in the dark for 20–30 min. After washing three times with PBS, fluorescence was observed using a Leica DMI 3000B fluorescence microscope. To further assess intercellular ROS levels, HaCaT cells were trypsinized, collected, and analyzed via flow cytometry (Cytomics FC500). Moreover, the intercellular ROS levels were quantified using a plate reader at excitation/emission wavelengths of 488/525 nm.

### HaCaT Cell Migration and Extracellular Matrix Formation

The effect of MNA patches on HaCaT cell migration was evaluated using a scratch assay. Briefly, cells at a density of 1 × 10^6 ^cells per well were seeded in 6‐well plates and incubated at 37 °C until reaching 90% confluence. A scratch was then made in each well using a 200 µL pipette tip. Cells were treated with MNA0, MNA1, and MNA2 patches, which were placed in transwells, and incubated for an additional 5 days in Opti‐MEMTM I reduced serum medium. Untreated cells served as the control (TCP). All experiments were performed in triplicate. Microscopic images were captured on days 0,1, 3, and 5 using a Leica DM IL LED microscope (Wetzlar, Germany) to evaluate cell migration. The wound area in each image was quantified by measuring the cell‐free region using ImageJ image software. Finally, the scratch closure rate was calculated to quantify the cell migration based on Equation (6):^[^ ^87^ ^]^

(6) $$\mathrm{Scratch}\;\mathrm{closure}\;\mathrm{rate}\;\left(\%\right)=\frac{\left[A_{t0}-A_{t}\right]}{A_{t0}}\times 100$$

where *A_t_*_0_ and *A_t_* are the initial scratch areas, and after incubation with particles for specific time points.

Following treatment of HaCaT cells with various MNA patches for 3 and 5 days, collagen type I (Col I) deposition and TGF‐β1 expression were evaluated using quantitative polymerase chain reaction (qPCR). Total RNA was extracted using the innuMIX qPCR DSGreen Standard kit, according to the manufacturer's protocol. Reverse transcription was performed to synthesize complementary DNA (cDNA) using the RevertAid First Strand cDNA Synthesis Kit, according to the manufacturer's protocol. The qTOWER 2.0 system (Analytik Jena) was employed for the PCR reaction, and data were analyzed using qPCRsoft (Analytik Jena AG) software. The sequences of the primers used for the target genes are provided in Table S1 (Supporting Information). DNA amplification was performed under the following thermal cycling conditions: 2 min at 95 ± 3 °C, 40 cycles of 10 s at 95 °C and 30 s at 60 °C, and 1 cycle of 5 s/step at 65–95 °C. Gene expression of the Col I and TGF‐β1 was normalized to the housekeeping gene (Cyclophilin A, CypA) using the Δ*Ct* method (Δ*C_t_* = *C_t_*_gene_ − *C_t_*_CypA_). Finally, Relative gene expression levels were calculated using the comparative ΔΔ*C*_t_ method (ΔΔ*C_t_* = Δ*C_t_*_gene_ − Δ*C_t_*_control_), and the fold change in expression was determined using the 2^−∆∆Ct^.

### Flii siRNA‐Induced Knockdown Assessment

Flii siRNA‐induced knockdown and its effects on wound healing were investigated using the qPCR with Flii gene‐specific primers (Table S1, Supporting Information), according to the manufacturer's protocol (Bio‐Rad). After 3‐ and 5‐day treatment with various MNA patches, total RNA was extracted from HaCaT cells using the innuMIX qPCR DSGreen Standard kit (Analytik Jena), following the manufacturer's instructions. The mRNA levels of Flii were determined as mentioned in *“HaCaT cell migration and extracellular matrix formation”*. Untreated cells were used as the control.

To further assess Flii protein expression, immunohistochemical staining was performed according to the manufacturer's protocol (Invitrogen). Briefly, HaCaT cells were fixed with 3.5% PFA for 20 min and permeabilized with 0.1% Triton‐X‐100 for 5 min, at room temperature. Cells were then blocked in 10% BSA solution for 30 min. Next, a 1:400 dilution (0.25 µg mL^−1^) of Flii polyclonal primary antibody (diluted in 10% FBS) was added to the cells and incubated overnight at 4 °C. The samples were then washed three times with PBS. A 1:200 dilution of Alexa Fluor 488 goat anti‐rabbit IgG secondary antibody in 10% FBS was applied, followed by incubation in the dark for 40 min at room temperature. Nuclei were counterstained with DAPI (1:1000 dilution in PBS) for 5 min. Finally, the samples were imaged using a fluorescence microscope, and fluorescence intensity and localization were analyzed using Image J software.

### Immunomodulatory Property Evaluation

J774A.1 macrophages were seeded at a density of 4 × 10^5^ cells mL^−1^ in 6‐well plates. After reaching 70% confluency, the culture medium was replaced with serum‐free medium containing LPS (1 µg mL^−1^) to induce polarization toward the M1 phenotype and promote ROS generation, following the protocol previously published.^[^ ^85^ ^]^ After 24 h of LPS stimulation, the cells were gently rinsed with PBS and co‐cultured with MNA patches (MNA0, MNA1, and MNA2) placed in transwell inserts. The co‐cultures were maintained in 2 mL Opti‐MEM reduced serum medium and incubated at 37 °C for 1, 3, and 5 days. LPS‐stimulated macrophages cultured in Opti‐MEM reduced serum medium were used as the positive control, while unstimulated macrophages cultured without LPS or MNA treatment were used as the negative control. The following experiments were conducted to evaluate macrophage behavior.

### Cell Viability and Proliferation

To study the role of LPS treatment on the viability and proliferation of macrophages, live/dead (after 1 day) and Alamarblue (after 1, 3, and 5 days) assays were performed, respectively, according to the procedure mentioned in “*Cell viability and proliferation assay*”.

### NO Generation Evaluation

Nitric oxide (NO) production by J774A.1 macrophages was analyzed using the Griess assay, according to the instructions of Sigma Aldrich. The assay was performed after 1 and 3 days of culture. Following the incubation, the culture supernatants were collected, and the absorbance was measured at 540 nm using a microplate reader. NO levels were quantified based on nitrite concentration, calculated from a standard curve generated using sodium nitrate for each experiment. Macrophages cultured in DMEM containing 10% FBS without any treatments were used as the control.

### ROS Scavenging Evaluation

The intracellular ROS was also determined after 1 and 3 days of culture, similar to the procedure mentioned in “*In vitro antioxidation assay*”

The anti‐inflammatory properties of MNA patches were assessed by evaluating the expression levels of Arg‐1 (an M1 macrophage marker) and pro‐inflammatory cytokines TNF‐α and IL6 (associated with M2 markers), using q‐PCR. After 3 days of culture, total RNA was extracted using the innuMIX qPCR DSGreen Standard kit (Analytik Jena), following the manufacturer's instructions. First‐strand cDNA was synthesized via reverse transcription using the RevertAid First Strand cDNA Synthesis Kit according to the manufacturer's protocol. Finally, q‐PCR was performed to analyze gene expression levels with GAPDH used as the housekeeping gene for normalization. The relative mRNA expression levels were calculated as mentioned in “*HaCaT cell migration and extracellular matrix formation*”. The primer sequences for all target genes are provided in Table S1 (Supporting Information).

### In Vivo Wound Healing Evaluation

To establish a full‐thickness wound healing model, 12‐week‐old male Sprague–Dawley (SD) rats (*n* = 12, weight: 120 ± 20 g; Orient Bio; Seongnam, Korea) were used for subcutaneous implantation of MNA patches. During the study, animals were housed individually in temperature‐controlled cages (24 ± 1 °C) under a 12:12 light‐dark cycle, with free access to standard rat chow diet and water. All animal procedures were conducted under the Guidelines for the Care and Use of Laboratory Animals of Tehran University of Medical Sciences and were approved by the Animal Ethics Committee (Code of Ethics: IR.TUMS.AEC.1403.073).

An infected chronic wound model was established as previously described.^[^ ^32^ ^]^ Briefly, rats were anesthetized via intraperitoneal injection of Ketamine and xylazine. The dorsal hair was shaved using a clipper, and the skin surface was cleaned with 75% ethanol for disinfection. Full‐thickness, circular excisional wounds (1 cm in diameter) were created on the dorsum of each rat. To induce a chronic wound environment and minimize wound contraction, a plastic ring was sutured around the wound area using suture thread. This method maintains the wound's size and shape over time by limiting natural skin contraction. After 6 days of wound induction, the 12 rats were randomly divided into three treatment groups (*n* = 4): 1) chronic wounds treated with PBS (pH = 7.4), considered as control, 2) chronic wounds treated with MNA0 patches, and 3) chronic wounds treated with MNA1 patches. Immediately after anesthesia, the respective MNA patches were applied with gentle pressure to the wound sites for 5 min and then secured using Tegaderm film. Wound healing progression was documented by photographing the wounds on days 0, 3, 7, 10, and 14 using a digital camera. The remaining unhealed wound area was calculated using Equation (7):

(7) $$\mathrm{Wound}\;\mathrm{area}\;\left(\%\right)=\frac{A_{n}}{A_{0}}\times 100\%$$

where *A*_n_ represents the wound areas on days 3, 7, 10, and 14, and *A*_0_ represents the initial wound area on day 0. After 7 and 14 days, the rats were euthanized by administering an overdose of chloral hydrate, and the excised wound tissue was fixed in 10% formalin and subsequently embedded in paraffin for pathological slide preparation. Histological analysis was then performed as described in “*In vitro/ex vivo penetration experiments*”.

### Wound Microenvironment Examination

Hematoxylin‐eosin staining (H&E) and Masson's trichrome staining were used to evaluate intra‐tissue healing and collagen content. For H&E analysis, thin tissue sections were treated with hematoxylin for 3–8 min and 0.5% eosin for 6 min at room temperature. Following dehydration with alcohol (80% ethanol for 30 s, 95% ethanol for 1 min, 95% ethanol for 1 min, and absolute ethanol for 3 min), the H&E‐stained sections were imaged using a full slide scanner (Zeiss AxioScan.z1, Japan). The length of the neo‐epithelium and the thickness of the granulation tissue were determined and analyzed using ImageJ software. To assess collagen deposition in granulation tissue, hydrated tissue sections were stained with Masson's trichrome stain (Solarbio) following the manufacturer's instructions. The staining process involved sequential treatment with Weigert's iron hematoxylin solution, Biebrich scarlet acid fuchsin solution, and aniline blue solution. Finally, the stained samples were mounted for further analysis.

Immunohistochemical experiments were conducted using Col I (1:200), TGF‐β1 (1:200), IL6 (1:500), CD31 (1:200), and TNF‐α (1:500) antibodies. Briefly, after the wound sections were deparaffinized, rehydrated, and boiled in a citrate buffer at 100 °C, they were incubated with primary antibodies overnight at 4 °C. Then, the free primary antibodies were washed off with PBS, and the sections were incubated with goat‐anti‐rabbit IgG or goat‐anti‐mouse IgG antibodies at 37 °C for 1 h. The images of the samples were acquired and quantified using ImageJ software.

### Statistical Analysis

All numerical data were analyzed and presented as mean ± standard deviation (SD) based on a minimum of three independent experiments. Statistical assessments were conducted using GraphPad Prism 7.0 software (La Jolla, CA, USA), via one‐way or two‐way analysis of variance (ANOVA) followed by Tukey's post hoc test for multiple comparisons. A significance threshold of *p* < 0.05 was applied, with statistically significant differences marked in all graphical representations.

## Conflict of Interest

The authors declare no conflict of interest.

## Supporting information



Supporting Information

## Data Availability

The data that support the findings of this study are available from the corresponding author upon reasonable request.
