# Supplementary material for: ROS‐Scavenging Multifunctional Microneedle Patch Facilitating Wound Healing
Source: Adv Healthc Mater. 2025 Aug 20;14(31):e01886. doi: 10.1002/adhm.202501886 (PMC12683203; doi:10.1002/adhm.202501886)
Supplement: Supplementary file 1 — Supporting Information [file ADHM-14-0-s001.docx]

**Supporting Information**

**ROS‐Scavenging Multifunctional Microneedle Patch Facilitating Wound Healing**

*Mahshid Kharaziha*, Sahar Salehi*, Mahshid Shokri, Seyed Mohsen Ahmadi Tafti,* *Thomas Scheibel*

M. Kharaziha, M. Shokri

Department of Materials Engineering, Isfahan University of Technology, Isfahan 84156-83111, Iran

E-mail: kharaziha@iut.ac.ir

M. Ahmadi Tafti

Colorectal research center, Imam Khomeini hospital complex, Tehran University of medical sciences, Tehran, Iran

Division of colorectal surgery, Department of Surgery, Tehran University of Medical Sciences, Tehran, Iran

M. Kharaziha, S. Salehi, T. Scheibel

Department of Biomaterials, Faculty of Engineering Science, University of Bayreuth, Bayreuth 95447, Germany

E-mail: Sahar.Salehi@uni-bayreuth.de

T. Scheibel

Bayreuther Zentrum für Kolloide und Grenzflächen (BZKG), University of Bayreuth, Bayreuth 95447, Germany

Bayreuther Zentrum für Molekulare Biowissenschaften (BZMB), University of Bayreuth, Bayreuth 95447, Germany

Bayreuther Materialzentrum (BayMAT), University of Bayreuth, Bayreuth 95447, Germany

Bayerisches Polymerinstitut (BPI), University of Bayreuth, Bayreuth 95447, Germany

**Table 1.** Primer sequences used for RT-qPCR

| Gene | Company | Sequence (5’ → 3′) |
| --- | --- | --- |
| Arg-1, Mouse | Bio-Rad | Fwd: GTGAAGAACCCACGGTCTGT  Rev: CTGGTTGTCAGGGGAGTGTT |
| IL-6, Mouse | Bio-Rad | Fwd: CGATGATGCACTTGCAGAAA  Rev: TGGAAATTGGGGTAGGAAGG |
| TGF-β1, Mouse | Bio-Rad | Fwd: CTTCTCCACCAACTACTGCTTC  Rev: GGGTCCCAGGCAGAAGTT |
| TNF-α, Mouse | Bio-Rad | Fwd: CTGTAGCCCACGTCGTAGC- Rev: TTGAGATCCATGCCGTTG- |
| GAPDH, Mouse | Bio-Rad | Fwd: GGTCCTCAGTGTAGCCCAAG  Rev: AATGTGTCCGTCGTGGATCT |
| Flii, Human | Bio-Rad | Fwd: CCTCCTACAGCTAGCGGTTATCAAC  Rev: GCATGTGCTGGATATATACCTGGCAG |
| Col I, Human | Bio-Rad | Fwd: AATTAATCTCAACAAACCC  Rev: ATTTTTTGTGGTTGGGGAG |
| Cyclophilin A (CypA) | Bio-Rad | Fwd: GGTTGGATGGCAAGCATGTG  Rev: TGCTGGTCTTGCCATTCCTG |


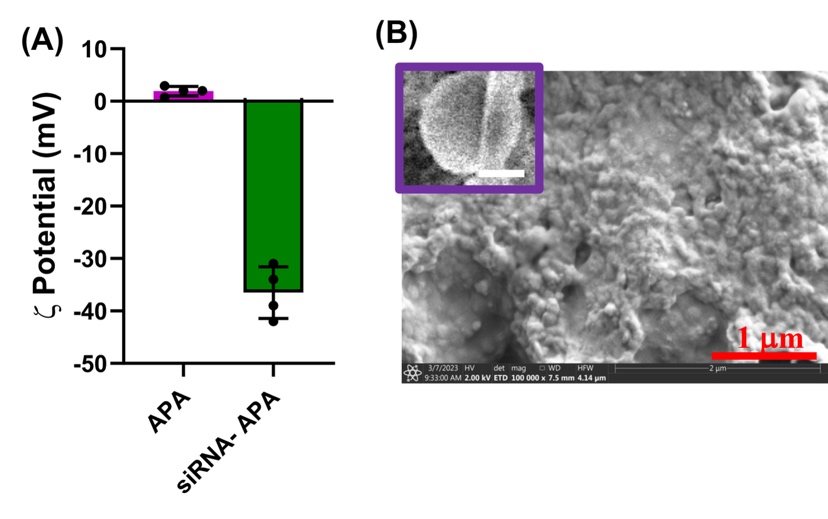


**Figure S1**. **Characterization of siRNA-laden APA particles:** (A) Zeta potential of APA particles, before and after siRNA loading. (C) SEM image of siRNA-laden APA particle


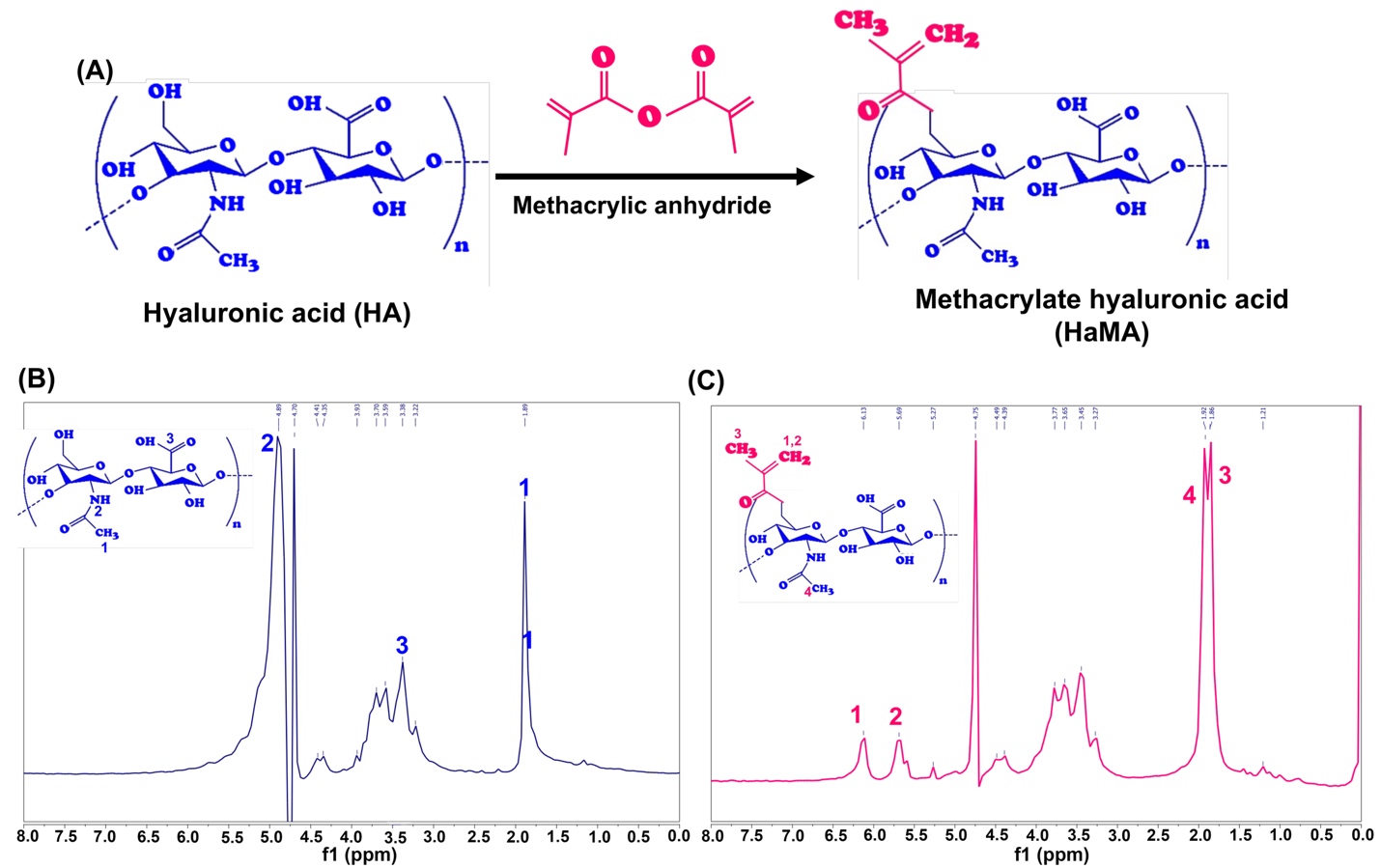


**Figure S2. Synthesis of HaMA polymer**: (A) The Schematic depiction of the synthetic route of HaMA synthesis, ^1^H NMR spectra of (B) HA and (C) HaMA.


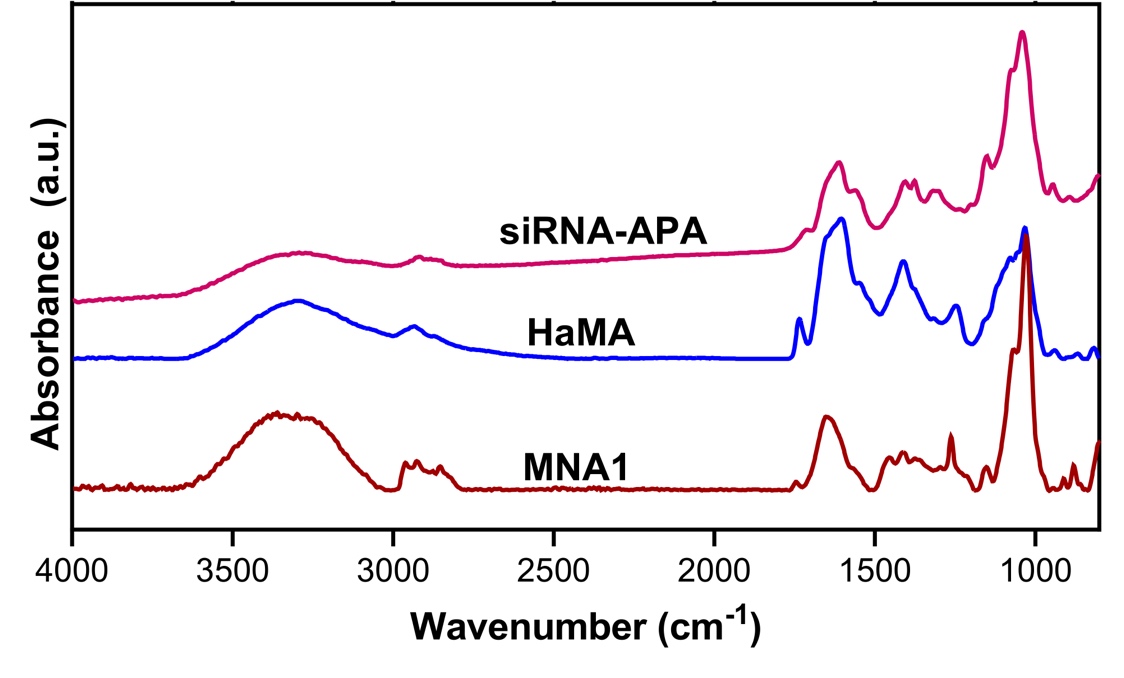


**Figure S3. Chemical characterization of base materials used in the fabrication of MNA patch**: FTIR spectrum of MNA1, HaMA and siRNA-APA particles.


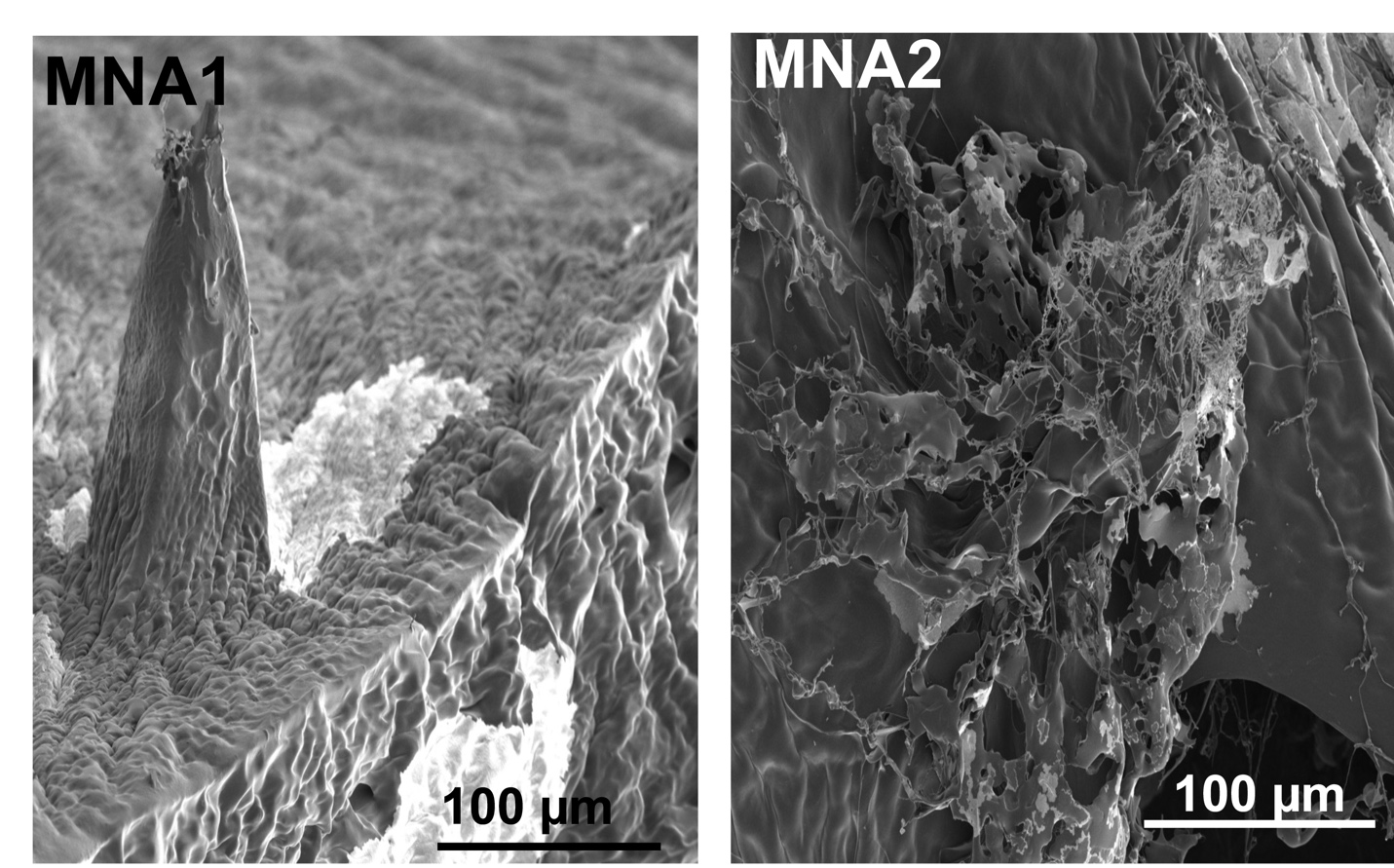


**Figure S4. Physiological stability of MNA patches:** SEM images of MNA1 and MNA2 patches after 14 days of soaking in PBS solution.

**
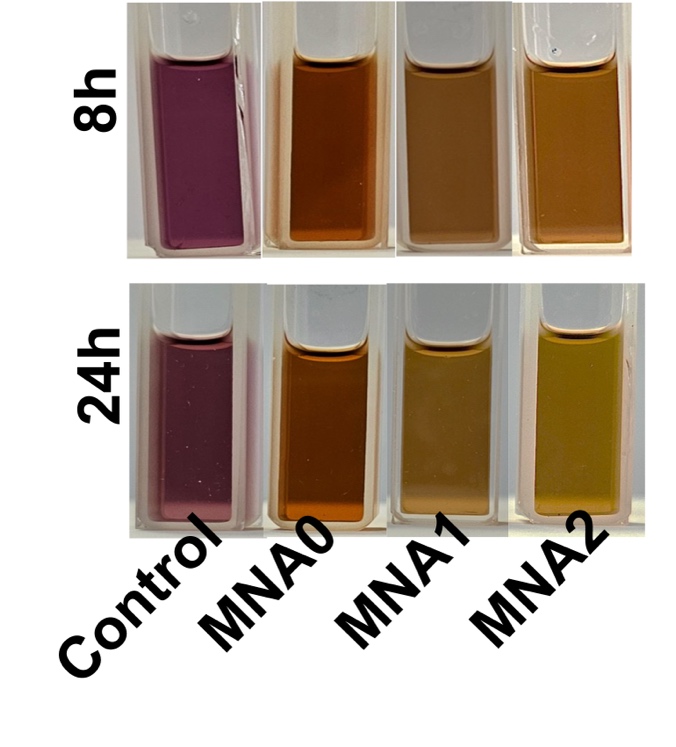
**

**Figure S5.** ROS scavenging property of MNA patches against 1-diphenyl-2-picrylhydrazyl (DPPH) radicals: digital images. The DPPH scavenging ability of samples was established by changing the color of the DPPH solution from purple to yellow.


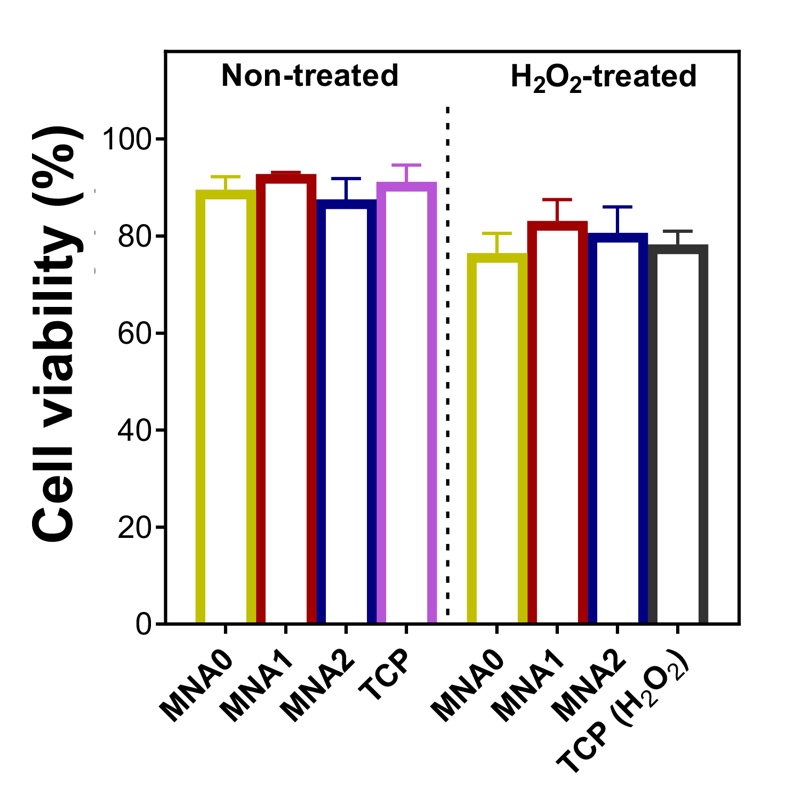


**Figure S6. The viability of HaCaT cells, before and after H_2_O_2_ treatment**, determined using a live/dead assay. The quantitative analysis of live and dead cells after 3 days of culture with and without H_2_O_2_ treatments. (**P*<0.05).


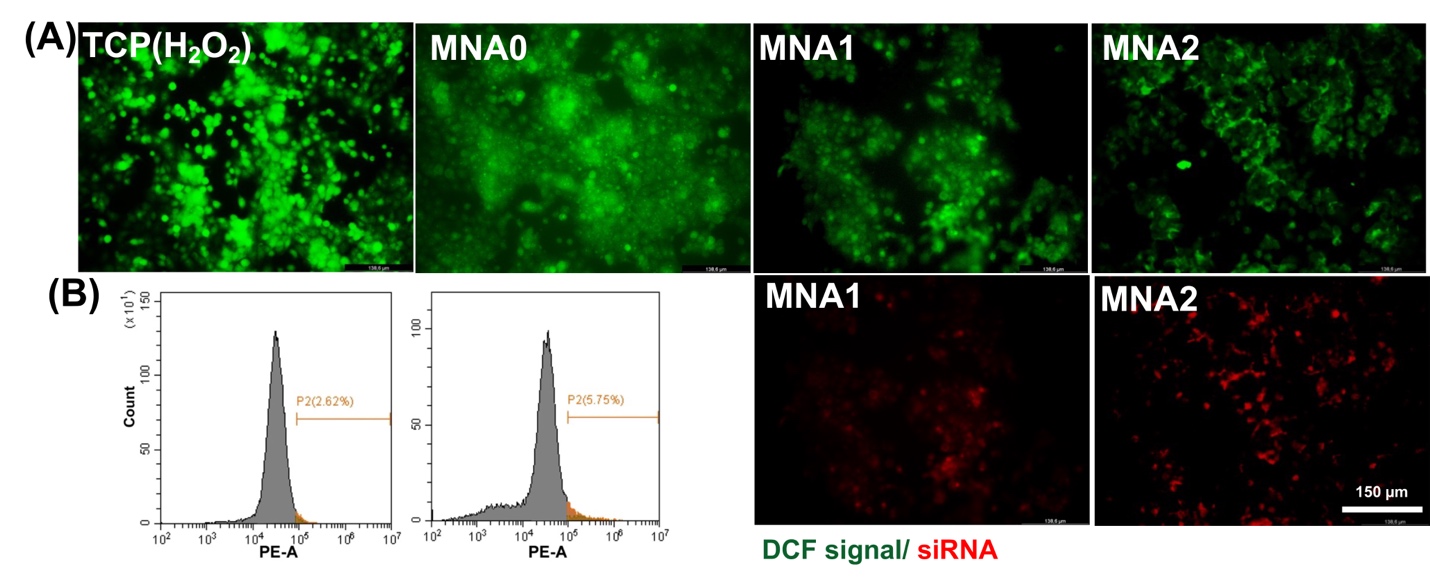


**Figure S7. Improvement of the microenvironment in HaCaT cells through antioxidative effects of MNA patches:** (A) Detection of intracellular ROS levels of HaCaT cells, after treatment with various MNA patches and incubation with H_2_O_2_ solution for 1 day. (B) Uptake of rhodamine B-labeled APA particles by HaCaT cells, assessed via fluorescence microscopy and flow cytometry after 1 day of culture. In the flow cytometry plot, the orange signal represents the proportion of cells that internalized rhodamine B-labeled APA particles.

**
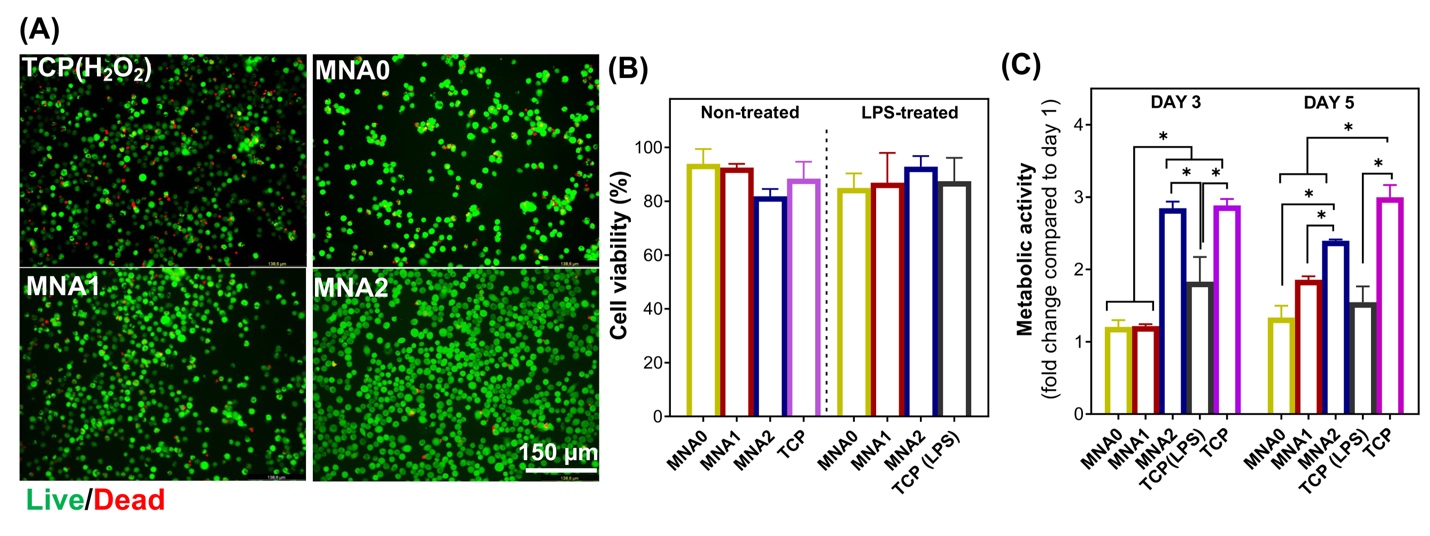
**

**Figure S8. Improvement of the microenvironment in J774.1 cells through antioxidative effects of MNA patches:** The viability of cells, before and after LPS treatment, determined using a live/dead assay: (A) Representative fluorescent images of live/dead staining after incubation with MNA patches and LPS (cells were stained with calcein-AM (green) and EthD-1(red) after a day of culture) and (B) quantitative analysis of live and dead cells before and after LPS treatment. (C) The effect of MNA patches on the metabolic activity of cells before and after LPS treatment assessed by Alamarblue^TM^ assay.


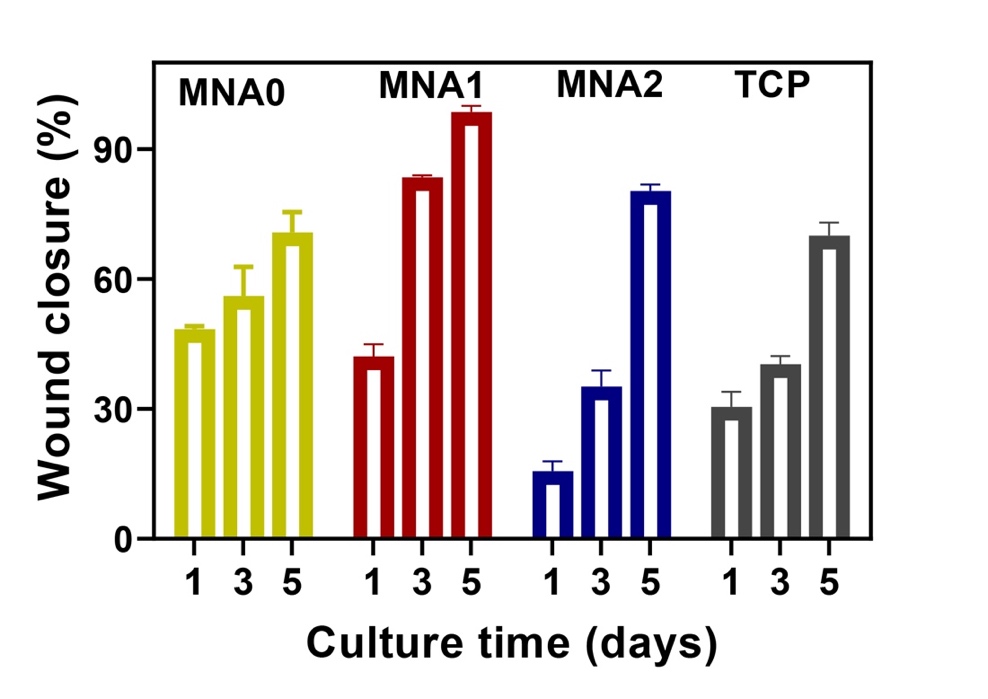


**Figure S9: *In vitro* wound healing mediated by MNA patches**: The scratch closure quantified as the percentage of the area that remains uncovered by cells.

**
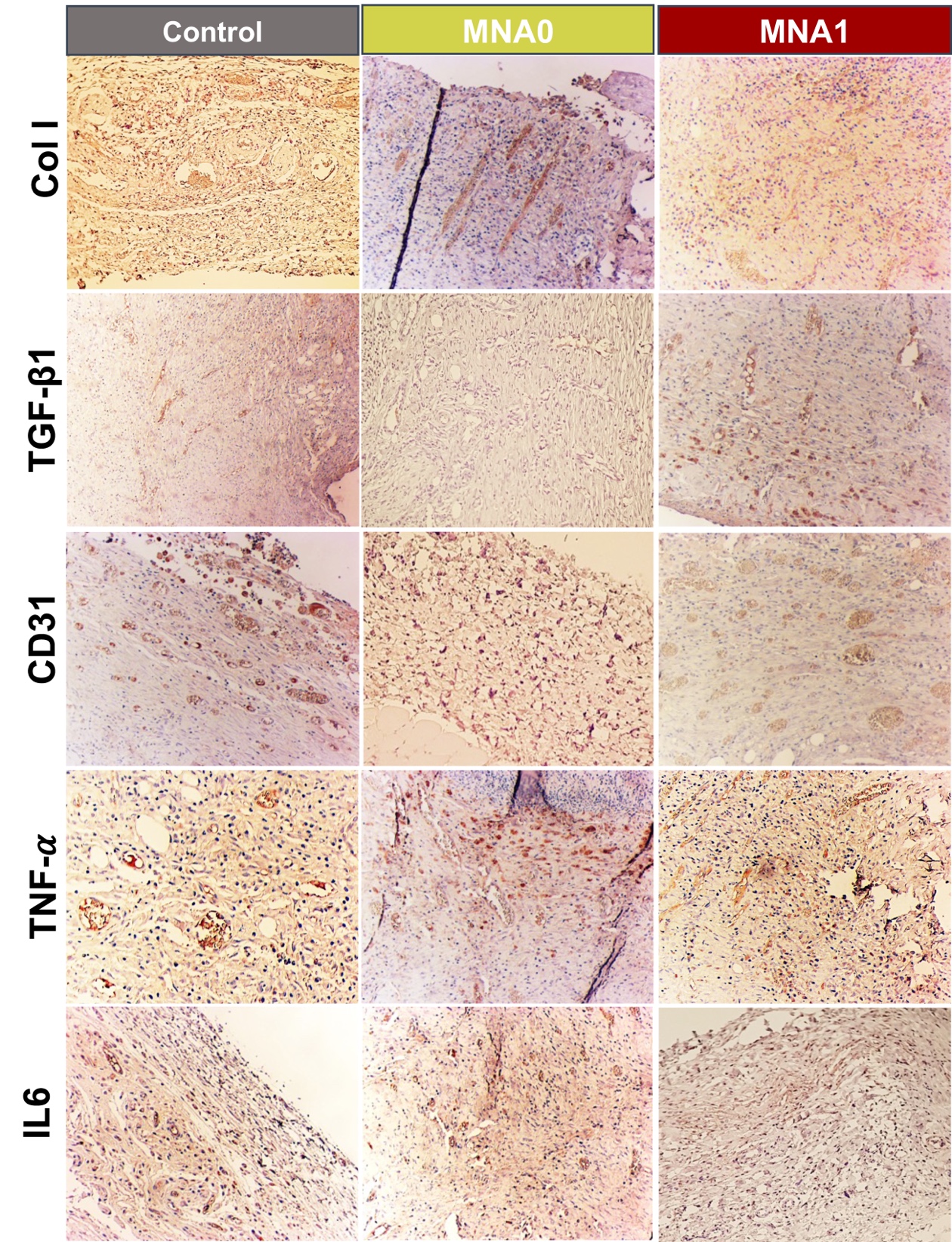
**

**Figure S10. Therapeutic outcome of MNA patch for tissue remodeling of the inflammatory wound in a rat model.** Immunohistochemistry (IHC) staining images of Col I, TGF-β1, CD31, IL-6, and TNF-α from the granulation tissues on day 7 of the wound healing process. All histology images were captured at 100× magnification.
